# Mobility-Aware ISAC-Assisted Cooperative Jamming for Secure Vehicular URLLC

**DOI:** 10.3390/s26154719

**Published:** 2026-07-24

**Authors:** Emmanouel T. Michailidis, Theodoros A. Tsiftsis, Nikolaos I. Miridakis

**Affiliations:** 1 Department of Informatics and Computer Engineering, University of West Attica, 12243 Aigaleo, Greece; nikozm@uniwa.gr; 2 Department of Informatics and Telecommunications, University of Thessaly, 35100 Lamia, Greece; tsiftsis@uth.gr

**Keywords:** cooperative jamming, integrated sensing and communication (ISAC), Kalman filtering, physical-layer security (PLS), ultra-reliable low-latency communication (URLLC), vehicular networks

## Abstract

This paper investigates vehicle-to-vehicle (V2V) integrated sensing and communication (ISAC) networks under ultra-reliable low-latency communication (URLLC) constraints in the presence of a vehicular eavesdropper (VE). To address the lack of instantaneous eavesdropper channel state information (CSI) in high-mobility scenarios, a vehicular jammer (VJ) is employed to perform radar-based sensing and extended Kalman filter (EKF)-based tracking of the VE’s kinematic state. The estimated state information and its posterior uncertainty are shared with the legitimate transmitter and are used to construct uncertainty-aware spatial covariance matrices for the VE-related channels. Based on these covariance matrices, the VJ transmits artificial noise (AN) in the null space of the legitimate receiver, thereby degrading the VE’s reception while avoiding interference to the intended link. In this context, a finite-blocklength (FBL) secrecy-rate framework is developed together with a two-time-scale optimization strategy, where the sensing resources are optimized at the slot level to enhance EKF tracking accuracy, while the transmit covariance and AN covariance matrices are optimized at the frame level to maximize the average FBL secrecy rate. The resulting non-convex problem is handled through semidefinite relaxation (SDR), alternating optimization (AO), and successive convex approximation (SCA). Simulation results show that the proposed framework improves secrecy robustness against mobility and sensing-induced spatial uncertainty.

## 1. Introduction

The rapid evolution of vehicular networks is driven by the need to support ultra-reliable low-latency communication (URLLC) for safety-critical applications, such as collision avoidance, cooperative driving, and autonomous navigation [1,2]. However, the broadcast nature of wireless communications inherently exposes vehicular networks to eavesdropping threats, especially in highly dynamic environments where malicious vehicles can act covertly as vehicular eavesdroppers (VEs) [3]. Physical-layer security (PLS) has been recognized as an effective approach to enhance confidentiality by leveraging the properties of the wireless channel [4]. Within this framework, cooperative jamming has been extensively explored, whereby dedicated jammers emit artificial noise (AN) to disrupt the eavesdropper’s reception while maintaining the performance of the legitimate link [5]. However, the efficiency of such techniques strongly depends on the availability of accurate information about the eavesdropper’s channel or spatial position, which is particularly difficult to obtain when VEs operate passively without transmitting pilots or feedback signals.

Meanwhile, integrated sensing and communication (ISAC) has recently emerged as a key enabling technology for vehicular networks, allowing communication nodes to simultaneously perform sensing and data transmission [6,7,8]. By exploiting radar-like sensing capabilities, ISAC enables legitimate vehicles to estimate and track the position, motion, and spatial characteristics of surrounding targets, including potential eavesdroppers [9]. Such sensing information can be leveraged to support direction-aware beamforming and AN transmission, thereby improving secrecy performance. However, in practical high-mobility vehicular environments, the sensed information is inherently imperfect due to measurement noise, mobility-induced prediction errors, and limited sensing resources. Consequently, the spatial information available for communication design becomes uncertain and time-varying, making robust secrecy-oriented transmission significantly more challenging.

### 1.1. Contributions

This paper addresses the problem of secure URLLC-enabled ISAC-assisted vehicular networks under high mobility. The main contributions are summarized as follows:

- **Mobility-Aware ISAC Secrecy Framework with Cooperative Jamming:** We develop a vehicle-to-vehicle (V2V) ISAC architecture in which a legitimate transmitter cooperates with a vehicular jammer (VJ) that performs radar-based sensing and extended Kalman filter (EKF)-based tracking of a passive VE. The proposed framework establishes a unified sensing-to-communication pipeline in which mobility-aware tracking directly drives the communication design. The VJ estimates the VE’s kinematic state and posterior uncertainty, shares this information with the transmitter through a reliable low-rate control link, and simultaneously transmits AN to enhance PLS.
- **EKF-Driven Uncertainty Propagation for Statistical Secrecy Analysis:** We propose a systematic uncertainty-propagation framework that transforms the EKF posterior tracking uncertainty into uncertainty-aware spatial covariance matrices for the VE-related channels. The resulting covariance model captures both dominant directional information and uncertainty-induced spatial spreading. These covariance matrices constitute the statistical channel state information (CSI) used for secrecy-oriented beamforming and AN design, thereby establishing a direct methodological link between mobility estimation, channel uncertainty, and finite-blocklength (FBL) secrecy analysis.
- **Sequential Two-Time-Scale Sensing–Communication Optimization and Performance Evaluation:** We formulate a two-time-scale optimization framework in which sensing resources are optimized at the slot level to satisfy EKF tracking-accuracy requirements, while the transmit covariance and null-space AN shaping covariance are optimized at the frame level to maximize the average FBL secrecy rate. The proposed sequential optimization architecture couples the sensing and communication layers through the uncertainty-aware channel statistics generated by the EKF, enabling adaptive secrecy transmission under mobility-induced channel uncertainty. The resulting non-convex communication problem is solved using semidefinite relaxation (SDR), alternating optimization (AO), and successive convex approximation (SCA), while the sensing problem is handled through a monotone bisection-based energy allocation approach. Simulation results demonstrate the effectiveness of the proposed framework.

### 1.2. Structure

The remainder of this paper is organized as follows. Section 2 reviews the related work and highlights the research gap. Section 3 introduces the system model, including the two-time-scale sensing-communication protocol, VE mobility tracking, and signal model. Section 4 presents the channel and sensing models, uncertainty-aware spatial covariance construction, and FBL secrecy-rate analysis. Section 5 formulates the sensing-communication optimization problem and develops the proposed solution framework. Simulation results are provided in Section 6, while Section 7 concludes the paper and discusses future research directions.

*Notations:* Scalars, vectors, and matrices are denoted by lowercase italic, lowercase bold, and uppercase bold symbols, respectively. The operators ${\left(\cdot \right)}^{T}$, ${\left(\cdot \right)}^{H}$, tr(·), det(·), and rank(·) denote transpose, Hermitian transpose, trace, determinant, and matrix rank, respectively. Moreover, the notation A⪰0 indicates that A is Hermitian positive semidefinite (PSD). The symbols E[·], CN(μ,Σ), and N(μ,Σ) denote expectation, circularly symmetric complex Gaussian distribution, and real Gaussian distribution, respectively. In addition, the identity matrix of dimension *N* is denoted by $I_{N}$, while 0 denotes the all-zero vector or matrix with appropriate dimensions. The notation |·| denotes the absolute value of a scalar, ∥·∥ (or ${∥\cdot ∥}_{2}$) the Euclidean (vector) norm, and ${∥\cdot ∥}_{F}$ the Frobenius norm. Furthermore, the operator diag(·) forms a diagonal matrix from its arguments. Besides, the notation ${\left[x\right]}^{+}≜\max\left\{x,0\right\}$ is used throughout the paper, and $Q^{-1}\left(\cdot \right)$ denotes the inverse Gaussian *Q*-function. The main variables and symbols used in this paper are summarized in Table 1.

## 2. Background

Existing relevant studies can be broadly classified into two major categories according to the solution methods employed to solve the underlying optimization problems. The first category relies on mathematical optimization techniques, including convex optimization, SDR, AO, SCA, block coordinate descent (BCD), and fractional programming, to optimize beamforming, resource allocation, sensing, and PLS. These approaches provide rigorous analytical formulations and high-quality solutions under well-defined mathematical models. However, their computational complexity may become prohibitive in large-scale and highly dynamic wireless environments, particularly when optimization problems are highly non-convex or require real-time adaptation. Consequently, an increasing number of studies have explored artificial intelligence (AI)-based approaches [10], such as deep reinforcement learning (DRL), federated learning (FL), and other data-driven optimization methods, to improve adaptability and reduce the online computational burden in complex wireless systems. The following subsections review representative works from these two research directions and position the proposed framework within the broader context of optimization-based secure URLLC-enabled and ISAC-assisted networks.

### 2.1. URLLC-Enabled ISAC-Assisted Networks

In recent years, various architectures and optimization strategies have been proposed for URLLC-enabled ISAC-assisted networks. In [11], an ISAC design supporting both periodic and sensing-triggered aperiodic traffic was introduced, where joint beamforming and scheduling enabled simultaneous sensing and transmission. This design was modeled as a Partially Observable Markov Decision Process (POMDP) capturing latency, reliability, and sensing uncertainty. In [12], an ISAC-enabled vehicle-to-everything (V2X) system was investigated with the objective of minimizing sensing latency, where the joint optimization of sensing repetitions, blocklength, and detection thresholds was addressed using a branch-and-bound method. The study in [13] investigated a multiple-input multiple-output (MIMO)-ISAC system under the FBL regime to characterize the sensing-communication trade-off, formulating a multi-objective problem that minimized Cramér-Rao bounds (CRBs) while maximizing communication rate, solved using BCD and SCA. The study in [14] introduced an ISAC framework integrated with mobile broadband reliable low-latency communication (mBRLLC), where extremely large antenna arrays enabled simultaneous multi-user communication and sensing. A worst-case rate maximization problem under URLLC and sensing constraints was solved using AO with convex and manifold optimization techniques. In [15], a beyond-diagonal reconfigurable intelligent surface (BD-RIS)-assisted ISAC system was proposed employing rate-splitting multiple access (RSMA) under imperfect CSI. A sum-rate maximization problem was formulated by jointly optimizing beamforming, RIS coefficients, and rate allocation under URLLC and sensing signal-to-clutter-plus-noise ratio (SCNR) constraints, and was addressed using a Meta-Proximal Policy Optimization (Meta-PPO)-based DRL framework.

### 2.2. Secure URLLC-Enabled Networks

Security-aware designs have become increasingly important in URLLC-enabled networks. In [16], a multiple-input single-output (MISO) scheme for Internet of Things (IoT) applications was proposed, where confidential transmission under multiple eavesdroppers was achieved via short-packet communications. A joint beamforming and AN scheme was developed to maximize the minimum secrecy rate under power constraints, and the resulting non-convex problem was solved using convex inner approximation and sequential convex programming (SCP). The work in [17] investigated a URLLC scenario for Industrial IoT (IIoT) systems, formulating a secure energy efficiency (SEE) maximization problem by jointly optimizing blocklength, pilot length, and transmit power under latency, reliability, and secrecy constraints, which was addressed using the Dinkelbach’s method. In [18], a full-duplex cooperative non-orthogonal multiple access (FD-CNOMA)-based URLLC scheme was proposed to enhance PLS via AN-assisted relaying, where a user simultaneously forwarded data and injected AN under residual self-interference. In this regard, joint blocklength and power allocation optimization was performed. The study in [19] proposed a transmission design for URLLC in mission-critical IoT scenarios, where channel training and data transmission were jointly optimized using a two-way discriminatory channel estimation (DCE) protocol with AN injection. An achievable effective secrecy rate (AESR) metric was introduced along with a tight analytical bound, and resource allocation was optimized via an iterative algorithm. In [20], an FL framework for vehicular communications was developed by integrating URLLC with Zero-Trust principles, demonstrating significant improvements in latency, throughput, and security through simulations based on real vehicular datasets.

### 2.3. Secure ISAC-Assisted Networks

At the same time, there has been a growing research interest in the design of secure communication schemes for ISAC-assisted networks. In [4], an ISAC framework for Internet of Vehicles (IoV) systems was proposed, accounting for sensing-communication coupling interference. A secrecy rate maximization problem was formulated by jointly optimizing transmission and sensing power under sensing accuracy and communication reliability constraints, and was solved using BCD and SCA. The work in [21] introduced an AN-assisted beamforming design to counter eavesdroppers without requiring CSI, formulating a max–min secrecy rate problem under beampattern and power constraints, which was handled via a quadratic transform and SCA-based approach. In addition, the work in [22] incorporated RSMA and active RIS to tackle eavesdropper spatial uncertainty, formulating a max-min ergodic secrecy rate problem jointly optimizing beamforming, AN, RIS coefficients, and sensing operations, solved via an AO framework combining SCA and majorization-minimization. In [23], a RIS-assisted secure ISAC architecture was proposed, where sensing targets were treated as potential eavesdroppers and artificial interference signals were jointly designed with communication beamforming. The secrecy-rate and sensing-power maximization problem was solved through fractional programming, SDR, and AO. A RIS-assisted cell-free ISAC scheme was proposed in [24] to enhance PLS through joint optimization of distributed ISAC beamforming and RIS phase shifts. The formulated weighted sum secrecy-rate maximization problem was solved using SDR and AO, while sensing functionality was exploited to detect potential eavesdroppers. Recently, the work in [25] proposed a robust RIS-assisted full-duplex ISAC framework that jointly optimized RIS phase shifts, transmit beamforming, AN covariance, uplink transmit powers, and receive combiners to maximize sensing performance under secrecy and power constraints using an AO/SCA-based algorithm. In [26], a sensing-aided secure communication framework was proposed, where a two-stage protocol first localized eavesdroppers and then performed secure transmission via robust beamforming. The work in [9] developed a sensing-assisted PLS framework that jointly optimized secrecy rate and eavesdropper estimation accuracy by minimizing the CRB, capturing the coupling between sensing and security. Secure ISAC beamforming with dedicated sensing signals was studied in [27], formulating a sum-rate maximization problem under secrecy and sensing constraints and showing that only a limited number of sensing beams is required. In [28], a unified optimization framework was proposed addressing secrecy outage constraints and multiple sensing metrics via a generalized rate maximization formulation, solved using a gradient-based AO algorithm.

### 2.4. Research Gap

Despite the growing body of literature, as summarized in Table 2, several important limitations remain. First, many existing works rely on asymptotic Shannon-capacity formulations, which fail to capture the FBL nature of URLLC transmissions. Second, secure communication designs typically assume perfect or quasi-static knowledge of the eavesdropper’s channel, which is unrealistic in high-mobility vehicular environments, where only noisy and time-varying sensing-based geometric information is available. Third, although ISAC frameworks leverage sensing to enhance communication, sensing resources are usually treated as fixed, overlooking the fundamental tradeoff between sensing accuracy and communication efficiency under latency constraints. Fourth, mobility-aware modeling is largely neglected, as most prior works assume static or independently distributed eavesdroppers, ignoring temporal correlation, tracking errors, and prediction uncertainty. To the best of our knowledge, no prior work jointly integrates FBL-based secrecy modeling, mobility-aware tracking, uncertainty propagation, and joint sensing-communication optimization in ISAC-enabled vehicular networks. This paper aims to bridge this gap. Specifically, the proposed framework introduces a unified methodology that systematically couples mobility-aware sensing with secrecy-oriented transmission. In this context, the posterior uncertainty obtained from EKF-based tracking is propagated into uncertainty-aware spatial covariance matrices, which serve as the statistical CSI for FBL secrecy analysis and the subsequent communication optimization. This establishes a direct link between sensing quality, channel uncertainty, and secrecy performance, enabling a sequential two-time-scale sensing-communication optimization framework that explicitly accounts for vehicular mobility and tracking uncertainty.

## 3. System Model

**Figure 1:**
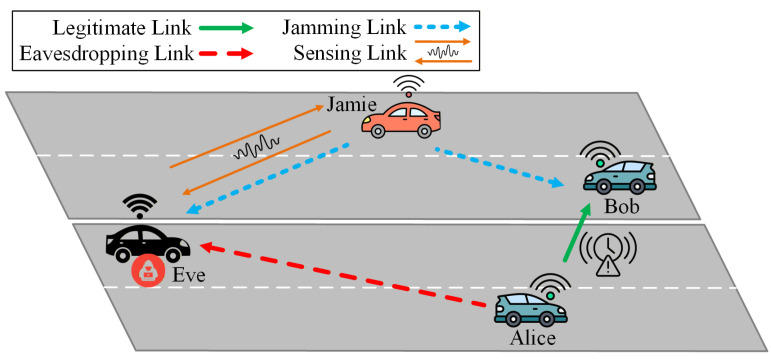
Illustration of the considered secure V2V ISAC scenario.

Consider the V2V ISAC scenario of Figure 1, where vehicles move along a multi-lane highway. A legitimate transmitter (Alice) sends confidential URLLC short-packet messages to a legitimate receiver (Bob) in the presence of a passive VE, denoted by Eve. To degrade Eve’s reception while minimizing interference to Bob, a cooperative VJ, denoted by Jamie, transmits AN. Alice, Bob, and Jamie are equipped with uniform linear arrays (ULAs) of $N_{A}$, $N_{B}$, and $N_{J}$ antennas, respectively, whereas Eve is equipped with a single antenna. We assume $N_{J}>N_{B}$ to facilitate effective null-space jamming. Each antenna element is omnidirectional, while directional transmission at the array level is achieved through beamforming and AN precoding. In the proposed ISAC architecture, Jamie performs radar-based sensing to detect and track Eve. Rather than integrating sensing and communication within a single transmitter, the proposed distributed ISAC architecture assigns sensing to Jamie and communication to Alice. This avoids additional sensing-resource consumption at the legitimate transmitter, allowing Alice to satisfy stringent latency and reliability requirements while Jamie simultaneously performs active sensing and cooperative jamming. The estimated state information is shared with Alice via a low-rate control link that is assumed to be error-free, reliable, and associated with negligible signaling delay relative to the slot duration. Since only the low-dimensional EKF posterior state estimate and the corresponding error covariance matrix are exchanged once per time slot, the associated signaling overhead is minimal, rendering the control-link latency negligible with respect to the adopted optimization framework. Nevertheless, in practical vehicular environments, packet losses, transmission errors, or signaling delays may result in outdated or inaccurate tracking information, thereby degrading the quality of the uncertainty-aware covariance matrices and consequently reducing the achievable secrecy performance. Extending the proposed framework to explicitly account for non-ideal control signaling constitutes an important direction for future work.

**Figure 2:**
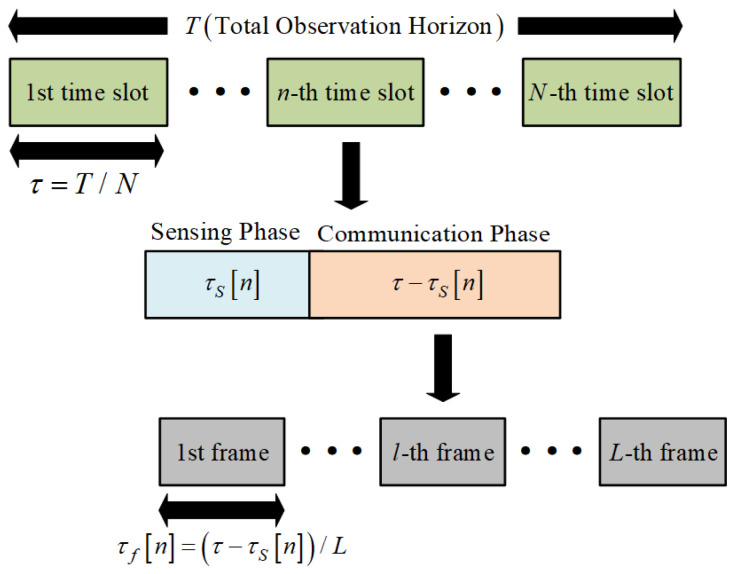
Two-time-scale sensing-communication protocol.

As shown in Figure 2, we adopt a two-time-scale model to capture the temporal dynamics of vehicular propagation, sensing updates, and URLLC transmission. The total observation horizon *T* is divided into *N* time slots, each of duration τ. The slot duration τ determines the update rate of ISAC-based sensing and mobility tracking. A smaller τ enables more frequent updates of Eve’s spatial information, enhancing robustness to mobility at the cost of increased sensing overhead. Conversely, a larger τ reduces sensing overhead but may result in outdated state and channel information. Within each slot n∈{1,…,N}, large-scale channel parameters (e.g., path loss) and slowly varying geometric characteristics (e.g., distance and angular direction) are assumed to be quasi-static, while small-scale fading varies across frames and Eve’s kinematic state evolves across slots. At the beginning of the *n*-th time slot, a duration $\tau_{S}\left[n\right]$ is allocated for radar-based sensing, which is performed exclusively by Jamie to estimate and track Eve’s geometric state. The remaining time, $\tau -\tau_{S}\left[n\right]$, is allocated for communication and divided into *L* frames, each of duration $\tau_{f}\left[n\right]=\left(\tau -\tau_{S}\left[n\right]\right)/L$. In each frame, Alice transmits one URLLC packet with $n_{c}$ channel uses. The packet transmission duration satisfies $n_{c}T_{\mathrm{sym}}\leq \tau_{f}\left[n\right]$, where $T_{\mathrm{sym}}$ is the symbol duration, ensuring that each packet fits within a frame. Thus, the slot-level time constraint is given by $\tau_{S}\left[n\right]+Ln_{c}T_{\mathrm{sym}}\leq \tau$. The transmission latency is constrained by $n_{c}T_{\mathrm{sym}}\leq \tau_{\max}^{\mathrm{trans}}$, where $\tau_{\max}^{\mathrm{trans}}$ denotes the maximum allowable transmission duration under URLLC requirements.

### 3.1. Mobility-Aware State Tracking of the VE

To account for Eve’s mobility, a discrete-time nonlinear state-space model is adopted at the slot level. The kinematic state of Eve is defined in polar coordinates with respect to Jamie’s position as $x_{JE}\left[n\right]={\left[\theta_{JE}\left[n\right],\;d_{JE}\left[n\right],\;v_{JE}\left[n\right]\right]}^{T}$, where $\theta_{JE}\left[n\right]$ is the angular direction from Jamie to Eve, $d_{JE}\left[n\right]$ is the corresponding distance, and $v_{JE}\left[n\right]$ denotes the along-road (longitudinal) relative velocity of Eve with respect to Jamie. As illustrated in Figure 1, Eve and Jamie travel in opposite directions along the road. The along-road motion is modeled with respect to Jamie’s local horizontal reference axis, following the relative-motion model in [29]. Under this convention, the radar measures the line-of-sight (radial) velocity component, which is expressed as $v_{\mathrm{rad},JE}\left[n\right]=-v_{JE}\left[n\right]\cos\left(\theta_{JE}\left[n\right]\right)$. The same coordinate and sign convention is consistently adopted throughout the state-transition model, the radar measurement model, and the EKF Jacobian derivation. The state evolution is modeled as $x_{JE}\left[n+1\right]=f\left(x_{JE}\left[n\right]\right)+w_{JE}\left[n\right]$, where f(·) denotes the nonlinear state-transition function, and $w_{JE}\left[n\right]∼N\left(0,Q_{w}\right)$ represents the process noise capturing mobility uncertainty, with diagonal covariance matrix $Q_{w}$. Specifically, the state-transition equations are given by [29]

(1) $$\begin{array}{cc} \theta_{JE}\left[n+1\right]\; & =\theta_{JE}\left[n\right]+\frac{v_{JE}\left[n\right]\tau }{d_{JE}\left[n\right]}\sin\;\left(\theta_{JE}\left[n\right]\right)+w_{\theta }\left[n\right], \end{array}$$

(2) $$\begin{array}{cc} d_{JE}\left[n+1\right]\; & =d_{JE}\left[n\right]-v_{JE}\left[n\right]\tau \cos\;\left(\theta_{JE}\left[n\right]\right)+w_{d}\left[n\right], \end{array}$$

(3) $$\begin{array}{cc} v_{JE}\left[n+1\right]\; & =v_{JE}\left[n\right]+w_{v}\left[n\right], \end{array}$$

where $w_{\theta }\left[n\right]$, $w_{d}\left[n\right]$, and $w_{v}\left[n\right]$ are zero-mean Gaussian noise components.

At the beginning of each time slot, Jamie performs ISAC-enabled radar-based sensing and obtains noisy observations of Eve’s angular direction, distance, and Doppler-based radial velocity. Before each sensing operation, Jamie steers its directional sensing beam according to the EKF-predicted angular direction ${\hat{\theta }}_{JE}\left[n|n-1\right]$. Consequently, the sensing operation does not require perfect prior knowledge of Eve’s instantaneous position. The beamwidth is selected to cover the angular uncertainty characterized by the associated prediction covariance, thereby maintaining reliable target illumination under the considered mobility conditions. Accordingly, the effective radar SNR benefits from the array gain provided by beam steering, whereas larger prediction uncertainties may increase the pointing mismatch and reduce the sensing accuracy. After receiving the radar echoes, the EKF measurement update refines the state estimate, thereby establishing a recursive closed-loop sensing procedure in which the sensing beam is continuously adapted according to the predicted target motion and the updated uncertainty information. The measurement vector is defined as $y_{JE}\left[n\right]={\left[{\hat{\theta }}_{JE}\left[n\right],\;{\hat{d}}_{JE}\left[n\right],\;{\hat{v}}_{\mathrm{rad},JE}\left[n\right]\right]}^{T}$, where ${\hat{d}}_{JE}\left[n\right]$ denotes the noisy distance measurement, while ${\hat{v}}_{\mathrm{rad},JE}\left[n\right]$ is the noisy measurement of the radial velocity component $-v_{JE}\left[n\right]\cos\left(\theta_{JE}\left[n\right]\right)$. The measurement model is expressed as $y_{JE}\left[n\right]=h\left(x_{JE}\left[n\right]\right)+v_{JE}\left[n\right]$, where

(4) $$h\left(x_{JE}\left[n\right]\right)=\left[\begin{array}{c} \theta_{JE}\left[n\right]\\ d_{JE}\left[n\right]\\ -v_{JE}\left[n\right]\cos\left(\theta_{JE}\left[n\right]\right) \end{array}\right].$$

The term $v_{JE}\left[n\right]∼N\left(0,R_{JE}\left[n\right]\right)$ represents the measurement noise with covariance matrix $R_{JE}\left[n\right]$ given by [30]

(5) $$R_{JE}\left[n\right]=\mathrm{diag}\left(\sigma_{\theta ,\mathrm{meas}}^{2}\left[n\right],\;\sigma_{d,\mathrm{meas}}^{2}\left[n\right],\;\sigma_{v,\mathrm{meas}}^{2}\left[n\right]\right),$$

where $\sigma_{\theta ,\mathrm{meas}}^{2}\left[n\right]$, $\sigma_{d,\mathrm{meas}}^{2}\left[n\right]$, and $\sigma_{v,\mathrm{meas}}^{2}\left[n\right]$ are the measurement-noise variances of the angle, distance, and radial velocity estimates, respectively. Following radar-based sensing and communication-assisted beam prediction models [29,30], the measurement-noise variances exhibit an inverse-SNR behavior, where the estimation accuracy improves with increasing sensing transmit power, matched-filtering gain, and sensing-channel strength. Specifically, the measurement-noise variances can be expressed as [29,30]

(6) $$\sigma_{\xi ,\mathrm{meas}}^{2}\left[n\right]=\frac{a_{\xi }^{2}\sigma_{r}^{2}}{G_{\xi }\left[n\right]\kappa^{2}{\left|\beta_{JE}^{\mathrm{rad}}\left[n\right]\right|}^{2}{\left|\delta_{JE}\left[n\right]\right|}^{2}P_{S,J}\left[n\right]},$$

where ξ∈{θ,d,v}, $a_{\xi }$ is an estimator-dependent constant, $\sigma_{r}^{2}$ is the radar receiver noise power, $G_{\xi }\left[n\right]=N_{\mathrm{sym}}\left[n\right]$ is the matched-filtering/processing gain [29], $N_{\mathrm{sym}}\left[n\right]$ is the number of sensing symbols used within the sensing phase of the *n*-th slot, $\kappa =\sqrt{N_{J}}$ is the coherent transmit array-gain factor, $\beta_{JE}^{\mathrm{rad}}\left[n\right]$ denotes the equivalent complex-valued radar-channel amplitude coefficient under two-way propagation between Jamie and Eve, $\delta_{JE}\left[n\right]$ represents the beam-steering alignment coefficient between Jamie’s sensing beam and the direction toward Eve, and $P_{S,J}\left[n\right]$ denotes Jamie’s sensing transmit power. For a fixed sensing symbol duration $T_{\mathrm{sym},S}$, it follows that $N_{\mathrm{sym}}\left[n\right]\approx \tau_{S}\left[n\right]/T_{\mathrm{sym},S}$. Based on a two-way radar propagation model, we obtain $\beta_{JE}^{\mathrm{rad}}\left[n\right]=\epsilon_{JE}\left[n\right]/{\left(2d_{JE}\left[n\right]\right)}^{2}$[30], where $\epsilon_{JE}\left[n\right]$ captures the radar cross-section (RCS) and scattering effects.

Due to the nonlinear state-transition model, an EKF is employed at Jamie, which shares the posterior state estimate and covariance with Alice for communication design. Let ${\hat{x}}_{JE}\left[n|n-1\right]$ and ${\hat{x}}_{JE}\left[n|n\right]$ denote the predicted and posterior state estimates, respectively, and let P[n|n−1] and P[n|n] denote the corresponding error covariance matrices. The EKF recursion proceeds as follows [29,30,31]:

*Prediction step:* The prior state estimate and the corresponding error covariance are propagated from the previous time slot, respectively, as

(7) $$\begin{array}{cc} {\hat{x}}_{JE}\left[n|n-1\right] & =f\left({\hat{x}}_{JE}\left[n-1|n-1\right]\right), \end{array}$$

(8) $$\begin{array}{cc} P[n|n-1] & =F\left[n-1\right]P\left[n-1|n-1\right]F^{T}\left[n-1\right]+Q_{w}. \end{array}$$

Here, $F\left[n-1\right]={\left.\frac{\partial f(x)}{\partial x}\right|}_{x={\hat{x}}_{JE}\left[n-1|n-1\right]}$ is the Jacobian of the state-transition function given by

(9) $$\begin{array}{cc} F[n-1]\; & =\left[\begin{array}{ccc} a_{1} & a_{2} & a_{3}\\ a_{4} & 1 & a_{5}\\ 0 & 0 & 1 \end{array}\right], \end{array}$$

where

(10) $$\begin{array}{cc} a_{1} & =1+\frac{{\hat{v}}_{JE}\left[n-1|n-1\right]\tau }{{\hat{d}}_{JE}\left[n-1|n-1\right]}\cos\;\left({\hat{\theta }}_{JE}\left[n-1|n-1\right]\right), \end{array}$$

(11) $$\begin{array}{cc} a_{2} & =-\frac{{\hat{v}}_{JE}\left[n-1|n-1\right]\tau }{{\hat{d}}_{JE}^{2}\left[n-1|n-1\right]}\sin\;\left({\hat{\theta }}_{JE}\left[n-1|n-1\right]\right), \end{array}$$

(12) $$\begin{array}{cc} a_{3} & =\frac{\tau }{{\hat{d}}_{JE}\left[n-1|n-1\right]}\sin\;\left({\hat{\theta }}_{JE}\left[n-1|n-1\right]\right), \end{array}$$

(13) $$\begin{array}{cc} a_{4} & ={\hat{v}}_{JE}\left[n-1|n-1\right]\tau \sin\;\left({\hat{\theta }}_{JE}\left[n-1|n-1\right]\right), \end{array}$$

(14) $$\begin{array}{cc} a_{5} & =-\tau \cos\;\left({\hat{\theta }}_{JE}\left[n-1|n-1\right]\right). \end{array}$$

*Measurement linearization:* The measurement function is nonlinear. Hence, the Jacobian H[n] is evaluated at the predicted state ${\hat{x}}_{JE}\left[n|n-1\right]$ as follows

(15) $$\begin{array}{cc} H[n]\; & ={\left.\frac{\partial h(x)}{\partial x}\right|}_{x={\hat{x}}_{JE}\left[n|n-1\right]}=\left[\begin{array}{ccc} 1 & 0 & 0\\ 0 & 1 & 0\\ b_{1} & 0 & b_{2} \end{array}\right], \end{array}$$

where $b_{1}={\hat{v}}_{JE}\left[n|n-1\right]\sin\;\left({\hat{\theta }}_{JE}\left[n|n-1\right]\right)$ and $b_{2}=-\cos\;\left({\hat{\theta }}_{JE}\left[n|n-1\right]\right)$.

*Update step:* The EKF gain is computed as

(16) $$K\left[n\right]=P\left[n|n-1\right]H^{T}\left[n\right]{\left(H\left[n\right]P\left[n|n-1\right]H^{T}\left[n\right]+R_{JE}\left[n\right]\right)}^{-1}$$

and the posterior state estimate and covariance matrix are updated, respectively, as

(17) $${\hat{x}}_{JE}\left[n|n\right]={\hat{x}}_{JE}\left[n|n-1\right]+K\left[n\right]\left(y_{JE}\left[n\right]-h\left({\hat{x}}_{JE}\left[n|n-1\right]\right)\right),$$

(18) $$\begin{array}{cc} P[n|n]\; & =\left(I_{3}-K\left[n\right]H\left[n\right]\right)P\left[n|n-1\right]. \end{array}$$

The posterior angular uncertainty at Jamie is given by $\sigma_{\theta ,JE}^{2}\left[n\right]={\left[P\left[n|n\right]\right]}_{1,1}$. To obtain the corresponding angular uncertainty at Alice, we apply first-order error propagation through the nonlinear coordinate transformation from Jamie-centered polar coordinates to the Alice-centered frame. Let $P_{\mathrm{pos}}\left[n|n\right]$ denote the 2×2 submatrix of P[n|n] associated with the state variables $\left(\theta_{JE}\left[n\right],d_{JE}\left[n\right]\right)$. The angular variance at Alice is approximated as $\sigma_{\theta ,AE}^{2}\left[n\right]\approx J_{\theta_{AE}}\left[n\right]\;P_{\mathrm{pos}}\left[n|n\right]\;J_{\theta_{AE}}^{T}\left[n\right]$, where $J_{\theta_{AE}}\left[n\right]=\left[\frac{\partial \theta_{AE}\left[n\right]}{\partial \theta_{JE}\left[n\right]},\;\frac{\partial \theta_{AE}\left[n\right]}{\partial d_{JE}\left[n\right]}\right]{|}_{\left({\hat{\theta }}_{JE}\left[n|n\right],\;{\hat{d}}_{JE}\left[n|n\right]\right)}$. Given that Alice has a known position relative to Jamie, $\left(x_{AJ},y_{AJ}\right)$, the EKF-based Cartesian estimate of Eve is ${\hat{x}}_{E}\left[n\right]={\hat{d}}_{JE}\left[n|n\right]\cos\left({\hat{\theta }}_{JE}\left[n|n\right]\right)$ and ${\hat{y}}_{E}\left[n\right]={\hat{d}}_{JE}\left[n|n\right]\sin\left({\hat{\theta }}_{JE}\left[n|n\right]\right)$. By defining $\Delta x\left[n\right]={\hat{x}}_{E}\left[n\right]-x_{AJ}$ and $\Delta y\left[n\right]={\hat{y}}_{E}\left[n\right]-y_{AJ}$, Alice’s estimated angular direction toward Eve is then ${\hat{\theta }}_{AE}\left[n\right]=atan2\left(\Delta y[n],\;\Delta x[n]\right)$. Using the differential identity $d\theta_{AE}=\frac{\Delta x[n]\;d(\Delta y[n])-\Delta y[n]\;d(\Delta x[n])}{\Delta x^{2}\left[n\right]+\Delta y^{2}\left[n\right]}$, the required partial derivatives are obtained via the chain rule as

(19) $$\frac{\partial \theta_{AE}}{\partial \theta_{JE}}=\frac{\Delta x\left[n\right]{\hat{d}}_{JE}\left[n|n\right]\cos\;\left({\hat{\theta }}_{JE}\left[n|n\right]\right)}{\Delta x^{2}\left[n\right]+\Delta y^{2}\left[n\right]}+\frac{\Delta y\left[n\right]{\hat{d}}_{JE}\left[n|n\right]\sin\;\left({\hat{\theta }}_{JE}\left[n|n\right]\right)}{\Delta x^{2}\left[n\right]+\Delta y^{2}\left[n\right]},$$

(20) $$\begin{array}{cc} \frac{\partial \theta_{AE}}{\partial d_{JE}}\; & =\frac{\Delta x\left[n\right]\sin\left({\hat{\theta }}_{JE}\left[n|n\right]\right)-\Delta y\left[n\right]\cos\left({\hat{\theta }}_{JE}\left[n|n\right]\right)}{\Delta x^{2}\left[n\right]+\Delta y^{2}\left[n\right]}. \end{array}$$

Accordingly, under the small-angle uncertainty approximation, the angular directions are modeled as Gaussian random variables $\theta_{JE}\left[n\right]∼N\left({\hat{\theta }}_{JE}\left[n|n\right],\sigma_{\theta ,JE}^{2}\left[n\right]\right)$ and $\theta_{AE}\left[n\right]∼N\left({\hat{\theta }}_{AE}\left[n\right],\sigma_{\theta ,AE}^{2}\left[n\right]\right)$.

### 3.2. Signal Model

Alice transmits the information-bearing signal $x_{A}\left[n,l\right]=w\left[n,l\right]s\left[n,l\right]$, where $w\left[n,l\right]\in C^{N_{A}\times 1}$ is the transmit beamforming vector and s[n,l]∼CN(0,1) is a unit-power information symbol. The transmit power at Alice is $E[∥x_{A}{\left[n,l\right]∥}^{2}{]=∥w\left[n,l\right]∥}^{2}$, subject to ${∥w\left[n,l\right]∥}^{2}\leq P_{A}$, where $P_{A}$ is Alice’s maximum transmit power per frame. Jamie transmits AN $x_{J}\left[n,l\right]=V\left[n,l\right]z\left[n,l\right]$, where $V\left[n,l\right]\in C^{N_{J}\times d_{J}}$ is the AN precoding matrix with $1\leq d_{J}\leq N_{J}$, and $z\left[n,l\right]∼\mathrm{CN}\left(0,I_{d_{J}}\right)$. The transmit power at Jamie is $E[∥x_{J}\left[n,l\right]{∥}^{2}]=\mathrm{tr}\left(V\left[n,l\right]V^{H}\left[n,l\right]\right)$, subject to $\mathrm{tr}\left(V\left[n,l\right]V^{H}\left[n,l\right]\right)\leq P_{J}$, where $P_{J}$ is Jamie’s maximum AN transmit power per frame. To suppress interference toward Bob, the AN precoder is designed in the right null space of the Jamie-Bob MIMO channel $H_{JB}\left[n,l\right]\in C^{N_{B}\times N_{J}}$. Assuming perfect instantaneous CSI of $H_{JB}\left[n,l\right]$, full row rank, and $N_{J}>N_{B}$, the available null-space dimension satisfies $N_{J}-\mathrm{rank}\left(H_{JB}\left[n,l\right]\right)\geq d_{J}$. The legitimate Alice-Bob and Jamie-Bob channels are assumed to be perfectly known through conventional pilot-assisted channel estimation and channel reciprocity (or feedback). This idealized assumption enables the proposed framework to isolate the impact of the joint sensing-communication design, EKF-assisted mobility-aware tracking, and uncertainty-aware covariance construction without introducing the additional complexity of legitimate CSI estimation errors. Accordingly, the reported results should be interpreted as a best-case performance benchmark under ideal legitimate CSI acquisition. Under these assumptions, the AN can be ideally projected onto the null space of the Jamie-Bob channel, thereby eliminating AN interference at Bob. In contrast, no instantaneous CSI is assumed for the passive VE. Instead, only uncertainty-aware second-order channel statistics obtained through ISAC-based sensing and EKF tracking are available for secrecy optimization. In practical high-mobility V2V environments, however, CSI imperfections and channel aging may introduce residual AN leakage toward Bob. Therefore, robust AN design under imperfect CSI is left for future investigation. The corresponding projection matrix is given by

(21) $$P_{JB,\mathrm{null}}\left[n,l\right]=I_{N_{J}}-H_{JB}^{H}\left[n,l\right]{\left(H_{JB}\left[n,l\right]H_{JB}^{H}\left[n,l\right]\right)}^{-1}H_{JB}\left[n,l\right].$$

Moreover, the AN precoder is constructed as $V\left[n,l\right]=P_{JB,\mathrm{null}}\left[n,l\right]\tilde{V}\left[n,l\right]$, which ensures $H_{JB}\left[n,l\right]V\left[n,l\right]=0$. Here, $\tilde{V}\left[n,l\right]\in C^{N_{J}\times d_{J}}$ denotes the AN shaping matrix.

To exploit sensing information, $\tilde{V}\left[n,l\right]$ can be aligned with the dominant eigen-directions of the uncertainty-aware spatial covariance matrix toward Eve, denoted by ${\hat{R}}_{JE}\left[n\right]\in C^{N_{J}\times N_{J}}$, whose eigendecomposition is ${\hat{R}}_{JE}\left[n\right]=U_{JE}\left[n\right]\Lambda_{JE}\left[n\right]U_{JE}^{H}\left[n\right]$. A practical AN design is to select $\tilde{V}\left[n,l\right]=U_{JE}^{\left(1:d_{J}\right)}\left[n\right]{\left(\Lambda_{JE}^{\left(1:d_{J}\right)}\left[n\right]\right)}^{1/2}$, where $U_{JE}^{\left(1:d_{J}\right)}\left[n\right]$ contains the dominant eigenvectors, aligning AN transmission with Eve’s most significant spatial directions. However, in the proposed optimization framework, the AN design is generalized by introducing the covariance matrix

(22) $$\begin{array}{cc} Q_{J}\left[n,l\right]\; & =V\left[n,l\right]V^{H}\left[n,l\right]\\ \; & =P_{JB,\mathrm{null}}\left[n,l\right]S_{J}\left[n,l\right]P_{JB,\mathrm{null}}^{H}\left[n,l\right], \end{array}$$

where $S_{J}\left[n,l\right]=\tilde{V}\left[n,l\right]{\tilde{V}}^{H}\left[n,l\right]⪰0$ is the AN shaping covariance matrix before null-space projection. Compared with fixed eigen-beam alignment, this formulation enables more flexible AN shaping under uncertainty.

Bob’s received signal is given by

(23) $$y_{B}\left[n,l\right]=H_{AB}\left[n,l\right]w\left[n,l\right]s\left[n,l\right]+H_{JB}\left[n,l\right]V\left[n,l\right]z\left[n,l\right]+n_{B}\left[n,l\right],$$

where $n_{B}\left[n,l\right]∼\mathrm{CN}\left(0,\sigma_{B}^{2}I_{N_{B}}\right)$. Due to the null-space design, $H_{JB}\left[n,l\right]V\left[n,l\right]=0$, and hence the AN does not interfere with Bob. Eve’s received signal is given by

(24) $$y_{E}\left[n,l\right]=h_{AE}^{H}\left[n,l\right]w\left[n,l\right]s\left[n,l\right]+h_{JE}^{H}\left[n,l\right]V\left[n,l\right]z\left[n,l\right]+n_{E}\left[n,l\right],$$

where $h_{AE}\left[n,l\right]\in C^{N_{A}\times 1}$ and $h_{JE}\left[n,l\right]\in C^{N_{J}\times 1}$ denote the Alice–Eve and Jamie–Eve channels, respectively, and $n_{E}\left[n,l\right]∼\mathrm{CN}\left(0,\sigma_{E}^{2}\right)$.

## 4. Channel Model, ISAC Sensing, and Performance Metric

In this paper, the V2V links are modeled using a clustered geometric channel model operating at 5.9 GHz [32]. The adopted propagation model captures key vehicular channel characteristics, including multipath clustering, angular dispersion, mobility-dependent geometric variation, and time-varying fading conditions. Sub-6 GHz vehicular systems offer important advantages over millimeter-wave (mmWave) systems, since they provide more favorable propagation conditions, lower blockage sensitivity, and longer channel coherence times, thereby effectively supporting sensing and localization functionalities [33].

For transmitter *i* and receiver *j*, where i,j∈{A,B,J} and i≠j, the MIMO channel $H_{ij}\left[n,l\right]\in C^{N_{j}\times N_{i}}$ is expressed as $H_{ij}\left[n,l\right]=\sqrt{\beta_{ij}\left[n\right]}\sum_{c=1}^{C_{ij}}\alpha_{c,ij}\left[n,l\right]a_{r,j}\left(\theta_{c,ij}^{r}\left[n\right]\right)a_{t,i}^{H}\left(\theta_{c,ij}^{t}\left[n\right]\right)$. Here, $\beta_{ij}\left[n\right]=\beta_{0}{\left(d_{0}/d_{ij}\left[n\right]\right)}^{\alpha }$ is the large-scale path-loss coefficient, where $\beta_{0}$ represents the channel gain at the reference distance $d_{0}=1$ m, and α is the path-loss exponent. Moreover, $C_{ij}$ is the number of propagation clusters, while the complex coefficients $\alpha_{c,ij}\left[n,l\right]∼\mathrm{CN}\left(0,1/C_{ij}\right)$ capture the small-scale fading effects. The angles $\theta_{c,ij}^{t}\left[n\right]$ and $\theta_{c,ij}^{r}\left[n\right]$ correspond to the angle of departure (AoD) and angle of arrival (AoA), respectively, and are considered quasi-static over each time slot. All azimuth angles are measured with respect to the positive *x*-axis. The ULAs are assumed to have their array broadside aligned with the positive *x*-axis. Hence, the geometric angles obtained from the EKF tracking model coincide with the array-domain AoD/AoA angles used in the channel model. The transmit steering vector $a_{t,i}\left(\theta \right)\in C^{N_{i}\times 1}$ is given by $a_{t,i}\left(\theta \right)={\left[1,\;e^{j\frac{2\pi d_{i}}{\lambda }\sin\left(\theta \right)},\;\ldots ,\;e^{j\frac{2\pi d_{i}}{\lambda }\left(N_{i}-1\right)\sin\left(\theta \right)}\right]}^{T}$, where λ and $d_{i}$ denote the carrier wavelength and inter-element spacing, respectively. The receive steering vector, denoted by $a_{r,j}\left(\theta \right)\in C^{N_{j}\times 1}$, is defined analogously. Since the array broadside is aligned with the positive *x*-axis, the angle θ represents the global azimuth angle in the considered Cartesian coordinate system. The steering vectors are normalized such that $∥a_{t,i}{\left(\theta \right)∥}^{2}=N_{i}$ and $∥a_{r,j}{\left(\theta \right)∥}^{2}=N_{j}$. Under independent zero-mean fading, the average channel power satisfies $E[∥H_{ij}\left[n,l\right]{∥}_{F}^{2}]=\beta_{ij}\left[n\right]N_{i}N_{j}$, since $\sum_{c=1}^{C_{ij}}E[|\alpha_{c,ij}{|}^{2}]=1$, ensuring that the total channel gain is independent of the number of clusters.

The MISO channel from transmitter i∈{A,J} to Eve is modeled as $h_{iE}\left[n,l\right]\in C^{N_{i}\times 1}$, where $h_{iE}\left[n,l\right]=\sqrt{\beta_{iE}\left[n\right]}\sum_{c=1}^{C_{iE}}\alpha_{c,iE}\left[n,l\right]a_{t,i}\left(\theta_{c,iE}^{t}\left[n\right]\right)$, with $\alpha_{c,iE}\left[n,l\right]∼\mathrm{CN}\left(0,1/C_{iE}\right)$. The sensing process is assumed to primarily capture the dominant specular component of the Eve-related channel, which carries the majority of the spatial energy. Without loss of generality, this component is associated with one cluster (e.g., c=1), whose direction corresponds to the angle from Jamie to Eve and is approximated by the EKF-based estimate, i.e., $\theta_{1,JE}^{t}\left[n\right]\approx {\hat{\theta }}_{JE}\left[n|n\right]$. The remaining clusters correspond to diffuse scattering components that are not explicitly tracked, and their aggregate effect is captured through statistical modeling of channel uncertainty.

The sensing operation at Jamie is modeled as a monostatic radar system. The radar channel matrix $G_{JE}\left[n\right]\in C^{N_{J}\times N_{J}}$ is represented by a dominant specular component as $G_{JE}\left[n\right]=\gamma_{JE}\left[n\right]a_{t,J}\left(\theta_{JE}^{\mathrm{dom}}\left[n\right]\right)a_{t,J}^{H}\left(\theta_{JE}^{\mathrm{dom}}\left[n\right]\right)$, where $\theta_{JE}^{\mathrm{dom}}\left[n\right]={\hat{\theta }}_{JE}\left[n|n\right]$ is the dominant reflection angle obtained from EKF tracking and $\gamma_{JE}\left[n\right]$ is a complex reflection coefficient that captures the radar cross-section (RCS), two-way path loss, and phase. Following the Swerling-I model [12], $\gamma_{JE}\left[n\right]$ is constant within each slot and varies independently across slots.

### 4.1. Uncertainty-Aware Spatial Covariance Construction

The transmit-side spatial covariance toward Eve is given by

(25) $$\left({\hat{R}}_{iE}\left[n\right]=\beta_{iE}\left[n\right]\;E_{\theta }\left[a_{t,i}\left(\theta \right)a_{t,i}^{H}\left(\theta \right)\right]\right),$$

where the expectation is taken over the angular distribution. The covariance construction focuses on angular uncertainty while averaging out small-scale fading effects. Using a first-order Taylor expansion around ${\hat{\theta }}_{iE}\left[n\right]$, the steering vector is approximated as $a_{t,i}\left(\theta \right)\approx {\hat{a}}_{iE}\left[n\right]+{\left.\frac{\partial a_{t,i}\left(\theta \right)}{\partial \theta }\right|}_{{\hat{\theta }}_{iE}\left[n\right]}\Delta \theta$, where ${\hat{a}}_{iE}\left[n\right]=a_{t,i}\left({\hat{\theta }}_{iE}\left[n\right]\right)$ and $\Delta \theta =\theta -{\hat{\theta }}_{iE}\left[n\right]$ are assumed to have zero mean with variance $\sigma_{\theta ,iE}^{2}\left[n\right]$. This yields $E_{\theta }\;\left[a_{t,i}\left(\theta \right)a_{t,i}^{H}\left(\theta \right)\right]\approx {\hat{a}}_{iE}\left[n\right]{\hat{a}}_{iE}^{H}\left[n\right]+\sigma_{\theta ,iE}^{2}\left[n\right]\;{\left.\frac{\partial a_{t,i}\left(\theta \right)}{\partial \theta }\right|}_{{\hat{\theta }}_{iE}\left[n\right]}{\left.\frac{\partial a_{t,i}^{H}\left(\theta \right)}{\partial \theta }\right|}_{{\hat{\theta }}_{iE}\left[n\right]}$. The first term represents the dominant rank-one spatial component associated with the estimated direction, whereas the second term captures angular-error-induced spatial spreading. Since the derivative-dependent term depends on the array geometry and nominal angle, we adopt a tractable isotropic diagonal-loading approximation $\sigma_{\theta ,iE}^{2}\left[n\right]{\left.\frac{\partial a_{t,i}\left(\theta \right)}{\partial \theta }\right|}_{{\hat{\theta }}_{iE}\left[n\right]}{\left.\frac{\partial a_{t,i}^{H}\left(\theta \right)}{\partial \theta }\right|}_{{\hat{\theta }}_{iE}\left[n\right]}\approx \sigma_{r,iE}^{2}\left[n\right]I_{N_{i}}$, where i∈{J,A} and $\sigma_{r,iE}^{2}\left[n\right]=c_{r}\sigma_{\theta ,iE}^{2}\left[n\right]$, with the regularization coefficient $c_{r}>0$ controlling the robustness level of the covariance model [34]. The coefficient $c_{r}$ determines the amount of isotropic uncertainty incorporated into the covariance model. Smaller values preserve stronger directional information around the estimated steering vector, whereas larger values increase the isotropic uncertainty component, yielding a more conservative but less directional covariance representation. Moreover, $\sigma_{r,iE}^{2}\left[n\right]$ represents the variance of the isotropic diagonal-loading component that captures residual angular uncertainty arising from sensing errors and diffuse scattering effects. The uncertainty-aware spatial covariance matrices are then approximated as ([34], Equation(13))

(26) $${\hat{R}}_{JE}\left[n\right]\approx \beta_{JE}\left[n\right]\left({\hat{a}}_{JE}\left[n\right]{\hat{a}}_{JE}^{H}\left[n\right]+\sigma_{r,JE}^{2}\left[n\right]I_{N_{J}}\right),$$

(27) $${\hat{R}}_{AE}\left[n\right]\approx \beta_{AE}\left[n\right]\left({\hat{a}}_{AE}\left[n\right]{\hat{a}}_{AE}^{H}\left[n\right]+\sigma_{r,AE}^{2}\left[n\right]I_{N_{A}}\right),$$

where ${\hat{a}}_{JE}\left[n\right]=a_{t,J}\left({\hat{\theta }}_{JE}\left[n\right]\right)$, ${\hat{a}}_{AE}\left[n\right]=a_{t,A}\left({\hat{\theta }}_{AE}\left[n\right]\right)$, $\sigma_{r,JE}^{2}\left[n\right]=c_{r}\sigma_{\theta ,JE}^{2}\left[n\right]$, and $\sigma_{r,AE}^{2}\left[n\right]=c_{r}\sigma_{\theta ,AE}^{2}\left[n\right]$. The resulting covariance matrices are Hermitian PSD. This approximation has a clear physical interpretation. When the angular uncertainty $\sigma_{\theta ,iE}^{2}\left[n\right]\to 0$, the corresponding covariance ${\hat{R}}_{iE}\left[n\right]$ reduces to a rank-one directional matrix determined by the estimated dominant spatial direction. As the angular uncertainty increases, the diagonal-loading term becomes larger, reflecting stronger uncertainty-induced spatial dispersion. Consequently, accurate sensing yields highly directional covariance matrices, whereas larger tracking uncertainty produces more diffuse spatial energy distributions.

### 4.2. FBL Secrecy Rate

For the Alice-Bob link, the instantaneous achievable rate under Gaussian signaling is given by

(28) $$C_{B}\left[n,l\right]=\log_{2}\det\;\left(I_{N_{B}}+\frac{1}{\sigma_{B}^{2}}H_{AB}\left[n,l\right]Q_{s}\left[n,l\right]H_{AB}^{H}\left[n,l\right]\right),$$

where $Q_{s}\left[n,l\right]≜w\left[n,l\right]w^{H}\left[n,l\right]$ is the transmit covariance matrix. At Eve, the instantaneous signal-to-interference-plus-noise ratio (SINR) is expressed as

(29) $$\gamma_{E}\left[n,l\right]=\frac{h_{AE}^{H}\left[n,l\right]Q_{s}\left[n,l\right]h_{AE}\left[n,l\right]}{h_{JE}^{H}\left[n,l\right]Q_{J}\left[n,l\right]h_{JE}\left[n,l\right]+\sigma_{E}^{2}},$$

where $Q_{J}\left[n,l\right]≜V\left[n,l\right]V^{H}\left[n,l\right]$ is the AN covariance matrix. Due to the passive nature of Eve, instantaneous CSI of the Eve-related channels is unavailable. Instead, Alice and Jamie rely on ISAC-based sensing and EKF tracking to obtain uncertainty-aware second-order channel statistics. Therefore, rather than using Eve’s instantaneous SINR directly, we adopt a second-order statistical SINR surrogate based on the available channel covariance information. Specifically, using the identity $E\;\left[h^{H}Qh\right]=\mathrm{tr}\;\left(Q\;E\;\left[hh^{H}\right]\right)$, together with the covariance approximation $R_{iE}\left[n\right]=E\;\left[h_{iE}\left[n,l\right]h_{iE}^{H}\left[n,l\right]\right]\approx {\hat{R}}_{iE}\left[n\right]$, where i∈{A,J}, Eve’s average received signal and jamming powers are approximated by $\mathrm{tr}\;\left(Q_{s}\left[n,l\right]{\hat{R}}_{AE}\left[n\right]\right)$ and $\mathrm{tr}\;\left(Q_{J}\left[n,l\right]{\hat{R}}_{JE}\left[n\right]\right)$, respectively. This yields the following second-order statistical SINR surrogate:

(30) $${\tilde{\gamma }}_{E}\left[n,l\right]=\frac{\mathrm{tr}\;\left(Q_{s}\left[n,l\right]{\hat{R}}_{AE}\left[n\right]\right)}{\mathrm{tr}\;\left(Q_{J}\left[n,l\right]{\hat{R}}_{JE}\left[n\right]\right)+\sigma_{E}^{2}}.$$

This surrogate provides a tractable design metric for secrecy-oriented beamforming and AN optimization under passive-Eve CSI uncertainty. Accordingly, Eve’s average achievable rate is approximated as

(31) $${\tilde{C}}_{E}\left[n,l\right]\approx \log_{2}\;\left(1+\frac{\mathrm{tr}(Q_{s}\left[n,l\right]{\hat{R}}_{AE}\left[n\right])}{\mathrm{tr}\left(Q_{J}\left[n,l\right]{\hat{R}}_{JE}\left[n\right]\right)+\sigma_{E}^{2}}\right).$$

Using (28) and (31), the achievable secrecy rate under FBL transmission is lower-bounded as [35]

(32) $$R_{s}^{\mathrm{lb}}\left[n,l\right]={\left[C_{B}\left[n,l\right]-{\tilde{C}}_{E}\left[n,l\right]-\sqrt{\frac{V_{B}\left[n,l\right]}{n_{c}}}\frac{Q^{-1}\left(\varepsilon_{B}\right)}{\ln2}-\sqrt{\frac{V_{E}\left[n,l\right]}{n_{c}}}\frac{Q^{-1}\left(\varepsilon_{s}\right)}{\ln2}\right]}^{+},$$

where $\varepsilon_{B}$ is the decoding error probability at Bob and $\varepsilon_{s}$ is the maximum allowable information leakage probability. The channel dispersion terms $V_{B}\left[n,l\right]$ and $V_{E}\left[n,l\right]$ quantify the stochastic variability of the information density in the FBL regime. For the Alice-Bob link, we obtain $V_{B}\left[n,l\right]=\mathrm{tr}\;\left(I_{N_{B}}-{\left(I_{N_{B}}+S_{B}\left[n,l\right]\right)}^{-2}\right)$, where $S_{B}\left[n,l\right]=\frac{1}{\sigma_{B}^{2}}H_{AB}\left[n,l\right]Q_{s}\left[n,l\right]H_{AB}^{H}\left[n,l\right]⪰0$. Let ${\left\{\lambda_{i}\right\}}_{i=1}^{N_{B}}$ denote the eigenvalues of $S_{B}\left[n,l\right]$, with $\lambda_{i}\geq 0$, then $V_{B}\left[n,l\right]=\sum_{i=1}^{N_{B}}\left(1-{\left(1+\lambda_{i}\right)}^{-2}\right),\;0\leq V_{B}\left[n,l\right]<N_{B}$. For Eve’s link, we obtain $V_{E}\left[n,l\right]=1-{\left(1+{\tilde{\gamma }}_{E}\left[n,l\right]\right)}^{-2}$ with $0\leq V_{E}\left[n,l\right]<1$. We adopt a worst-case approximation by replacing the dispersion terms with their upper bounds, i.e., $V_{B}\left[n,l\right]\leq N_{B}$ and $V_{E}\left[n,l\right]\leq 1$. This corresponds to a pessimistic assumption where Bob experiences maximum decoding variability, while Eve operates under favorable conditions that maximize potential information leakage. Thus, the secrecy rate is approximated as

(33) $$R_{s}^{\mathrm{lb},\mathrm{approx}}\left[n,l\right]={\left[C_{B}\left[n,l\right]-{\tilde{C}}_{E}\left[n,l\right]-\eta_{\mathrm{FBL}}\right]}^{+},$$

where

(34) $$\eta_{\mathrm{FBL}}=\sqrt{\frac{N_{B}}{n_{c}}}\frac{Q^{-1}\left(\varepsilon_{B}\right)}{\ln2}+\frac{Q^{-1}\left(\varepsilon_{s}\right)}{\sqrt{n_{c}}\ln2}.$$

## 5. Problem Formulation and Solution

In this section, we develop a sensing-communication framework to maximize secrecy performance that operates at two layers. In the *outer layer*, sensing resources at Jamie are optimized at the slot level to enhance the EKF-based tracking accuracy of Eve, thereby refining the uncertainty-aware covariance matrices ${\hat{R}}_{AE}\left[n\right]$ and ${\hat{R}}_{JE}\left[n\right]$. In the *inner layer*, given the sensing and tracking outcomes, the communication variables are optimized at the frame level. The proposed framework follows a sequential two-time-scale optimization strategy, in which sensing and communication are coupled through the uncertainty-aware channel statistics obtained from EKF-based tracking. Specifically, the sensing layer determines the tracking accuracy and the resulting spatial covariance matrices, which directly influence the communication optimization by providing the statistical CSI used for beamforming and AN design. Nevertheless, the proposed framework is not fully coupled, since the sensing and communication variables are not jointly optimized within a unified optimization problem. This constitutes a limitation of the current framework and motivates future research on fully coupled sensing-communication optimization.

### 5.1. Outer-Layer Sensing and Tracking Optimization

Given the prior estimate ${\hat{x}}_{JE}\left[n-1|n-1\right]$ and covariance P[n−1|n−1], the measurement-noise model in (5), combined with the EKF prediction and update equations, indicates that the posterior covariance P[n|n] is influenced by the sensing variables $\left(P_{S,J}\left[n\right],\tau_{S}\left[n\right]\right)$ through the measurement-noise covariance $R_{JE}\left[n\right]$. Considering that the effective sensing energy is defined as $E_{S}\left[n\right]=P_{S,J}\left[n\right]\tau_{S}\left[n\right]$, we formulate a minimum-energy sensing problem subject to a required estimation-accuracy level as follows:

(35a)(P1):minPS,J[n],τS[n]PS,J[n]τS[n](35b)                s.t.τS[n]+LncTsym≤τ,(35c)ncTsym≤τmaxtrans,(35d)0≤τS[n]≤τ,(35e)0≤PS,J[n]≤PS,Jmax,(35f)trP[n|n]≤δP,

where $P_{S,J}^{\max}$ is the maximum sensing transmit power of Jamie and $\delta_{P}$ is the maximum allowable posterior state-estimation error level. The constraint in (35b) enforces the slot-level time budget, the constraint in (35c) ensures that each packet satisfies latency requirements, the constraint in (35d) bounds the sensing duration within the slot interval, the constraint in (35e) captures the hardware limitations on sensing transmit power, and the constraint in (35f) links sensing resource allocation to the EKF estimation accuracy. Although the EKF state vector contains variables with different physical units (angle, distance, and velocity), the covariance trace is employed as a scalar engineering measure of the overall tracking uncertainty rather than as a direct physical sum of variances. Accordingly, the trace constraint may be viewed as a normalized (or equivalently weighted) tracking-error metric, where the individual state variables are interpreted after appropriate scaling according to their nominal tolerable estimation-error levels. For notational simplicity, this normalization is reflected through the design threshold $\delta_{P}$, while the compact expression $\mathrm{tr}\left(P\left[n|n\right]\right)\leq \delta_{P}$ is retained throughout the paper. The threshold $\delta_{P}$ is selected empirically through preliminary simulations to achieve the desired EKF tracking accuracy while maintaining a feasible sensing-resource allocation.

Since the measurement model is nonlinear, the EKF update is based on the linearized measurement matrix H[n] evaluated at ${\hat{x}}_{JE}\left[n|n-1\right]$. Thus, the posterior covariance can be written in the linearized information form as $P\left[n|n\right]={\left(P^{-1}\left[n|n-1\right]+H^{T}\left[n\right]R_{JE}^{-1}\left[n\right]H\left[n\right]\right)}^{-1}$. Using the sensing-dependent measurement-noise model, the inverse covariance matrix can be expressed as $R_{JE}^{-1}\left[n\right]=E_{S}\left[n\right]\beta_{JE}^{\mathrm{eff}}\left[n\right]D_{c}$, where $\beta_{JE}^{\mathrm{eff}}\left[n\right]=\kappa^{2}{\left|\beta_{JE}^{\mathrm{rad}}\left[n\right]\right|}^{2}{\left|\delta_{JE}\left[n\right]\right|}^{2}/\left(\sigma_{r}^{2}T_{\mathrm{sym},S}\right)$ is the effective sensing SNR per unit energy and $D_{c}=\mathrm{diag}\left(1/a_{\theta }^{2},\;1/a_{d}^{2},\;1/a_{v}^{2}\right)$. Accordingly, the posterior covariance matrix can be expressed as follows:

(36) $$\left(P\left[n|n\right]={\left(P^{-1}\left[n|n-1\right]+E_{S}\left[n\right]\beta_{JE}^{\mathrm{eff}}\left[n\right]H^{T}\left[n\right]D_{c}H\left[n\right]\right)}^{-1}\right).$$

Considering that $H^{T}\left[n\right]D_{c}H\left[n\right]⪰0$, the matrix inside the inverse increases monotonically with the sensing energy $E_{S}\left[n\right]$ in the PSD sense. Therefore, the posterior covariance matrix P[n|n] decreases monotonically as $E_{S}\left[n\right]$ increases, implying that tr(P[n|n]) is a monotone decreasing function of the sensing energy. As a result, the minimum required sensing energy can be efficiently obtained through a scalar monotone search procedure.

If the predicted covariance already satisfies $\mathrm{tr}\left(P\left[n|n-1\right]\right)\leq \delta_{P}$, then no sensing energy is required, and the optimal solution is $E_{S}^{★}\left[n\right]=0$. Otherwise, if the tracking constraint is feasible within the available sensing-energy budget, the optimal solution satisfies $\mathrm{tr}\left(P\left[n|n\right]\right)=\delta_{P}$. In this case, the required sensing energy $E_{S}^{★}\left[n\right]$ is obtained by solving the scalar equation $\mathrm{tr}\left(P\left[n|n\right]\right)=\delta_{P}$ via bisection, as summarized in Algorithm 1. After obtaining $E_{S}^{★}\left[n\right]$, the sensing duration and transmit power can be recovered from any feasible allocation satisfying $E_{S}^{★}\left[n\right]=P_{S,J}^{★}\left[n\right]\tau_{S}^{★}\left[n\right]$. To maximize the remaining communication time, we adopt the minimum-duration policy $\tau_{S}^{★}\left[n\right]=E_{S}^{★}\left[n\right]/P_{S,J}^{\max}$ and $P_{S,J}^{★}\left[n\right]=E_{S}^{★}\left[n\right]/\tau_{S}^{★}\left[n\right]$, provided that $\tau_{S}^{★}\left[n\right]+Ln_{c}T_{\mathrm{sym}}\leq \tau$. The resulting posterior covariance $P^{★}\left[n|n\right]$ is then used for same-slot uncertainty-aware covariance construction and inner-layer communication design.
**Algorithm 1** Outer-Layer Sensing Optimization1:**Input:** ${\hat{x}}_{JE}\left[n-1|n-1\right]$, P[n−1|n−1], $Q_{w}$, $\beta_{JE}^{\mathrm{eff}}\left[n\right]$, $D_{c}$, $\delta_{P}$, $P_{S,J}^{\max}$, τ, *L*, $n_{c}$, $T_{\mathrm{sym}}$, $\tau_{\max}^{\mathrm{trans}}$, and tolerance ϵ.2:Predict ${\hat{x}}_{JE}\left[n|n-1\right]$ and P[n|n−1].3:Set $M\left[n\right]=\beta_{JE}^{\mathrm{eff}}\left[n\right]H^{T}\left[n\right]D_{c}H\left[n\right]$, with H[n] evaluated at ${\hat{x}}_{JE}\left[n|n-1\right]$.4:**if** $n_{c}T_{\mathrm{sym}}>\tau_{\max}^{\mathrm{trans}}$ or $\tau -Ln_{c}T_{\mathrm{sym}}\leq 0$ **then**5:   Declare infeasible and stop.6:**end if**7:Set $E_{\min}=0$, $E_{\max}=P_{S,J}^{\max}\left(\tau -Ln_{c}T_{\mathrm{sym}}\right)$.8:**if **$\mathrm{tr}\left(P\left[n|n-1\right]\right)\leq \delta_{P}$** then**9:   $E_{S}^{★}\left[n\right]=0$, $P^{★}\left[n|n\right]=P\left[n|n-1\right]$.10:**else**11:   $P_{\max}\left[n|n\right]={\left(P^{-1}\left[n|n-1\right]+E_{\max}M\left[n\right]\right)}^{-1}$.12:   **if** $\mathrm{tr}\left(P_{\max}\left[n|n\right]\right)>\delta_{P}$ **then**13:     Declare infeasible and stop.14:   **end if**15:   **while** $E_{\max}-E_{\min}>\epsilon$ **do**16:     $E_{\mathrm{mid}}=\left(E_{\min}+E_{\max}\right)/2$.17:     $P_{\mathrm{mid}}\left[n|n\right]={\left(P^{-1}\left[n|n-1\right]+E_{\mathrm{mid}}M\left[n\right]\right)}^{-1}$.18:     If $\mathrm{tr}\left(P_{\mathrm{mid}}\left[n|n\right]\right)\leq \delta_{P}$, set $E_{\max}=E_{\mathrm{mid}}$; otherwise set $E_{\min}=E_{\mathrm{mid}}$.19:   **end while**20:   $E_{S}^{★}\left[n\right]=E_{\max}$, $P^{★}\left[n|n\right]={\left(P^{-1}\left[n|n-1\right]+E_{S}^{★}\left[n\right]M\left[n\right]\right)}^{-1}$.21:**end if**22:Recover $\tau_{S}^{★}\left[n\right]=E_{S}^{★}\left[n\right]/P_{S,J}^{\max}$ and $P_{S,J}^{★}\left[n\right]=P_{S,J}^{\max}$ if $E_{S}^{★}\left[n\right]>0$; otherwise set both to zero.23:**Output:** $E_{S}^{★}\left[n\right]$, $\tau_{S}^{★}\left[n\right]$, $P_{S,J}^{★}\left[n\right]$, $P^{★}\left[n|n\right]$.

### 5.2. Inner-Layer FBL-Aware Secrecy Maximization

Given the optimal sensing variables $\left\{P_{S,J}^{★}\left[n\right],\tau_{S}^{★}\left[n\right]\right\}$ and the resulting uncertainty-aware covariance matrices $\left\{{\hat{R}}_{AE}\left[n\right],{\hat{R}}_{JE}\left[n\right]\right\}$, we aim to optimize $Q_{s}\left[n,l\right]$ and $S_{J}\left[n,l\right]$ to maximize the average FBL secrecy rate. To ensure that the AN does not interfere with Bob, we enforce the null-space structure $V\left[n,l\right]=P_{JB,\mathrm{null}}\left[n,l\right]\tilde{V}\left[n,l\right]$, where $P_{JB,\mathrm{null}}\left[n,l\right]$ is the projection matrix onto the null space of the channel $H_{JB}\left[n,l\right]$. Let $S_{J}\left[n,l\right]≜\tilde{V}\left[n,l\right]{\tilde{V}}^{H}\left[n,l\right]⪰0$ denote the AN shaping covariance matrix, which leads to the AN covariance matrix $Q_{J}\left[n,l\right]=P_{JB,\mathrm{null}}\left[n,l\right]S_{J}\left[n,l\right]P_{JB,\mathrm{null}}^{H}\left[n,l\right]$. By defining $f\left(Q_{s}\left[n,l\right],S_{J}\left[n,l\right]\right)≜C_{B}\left(Q_{s}\left[n,l\right]\right)-{\tilde{C}}_{E}\left(Q_{s}\left[n,l\right],S_{J}\left[n,l\right]\right)-\eta_{\mathrm{FBL}}$, the inner-layer optimization problem is formulated as follows:

(37a)(P2):max{Qs[n,l],SJ[n,l]}1NL∑n=1N∑l=1LfQs[n,l],SJ[n,l](37b)s.t.trQs[n,l]≤PA,tr(PJB,null[n,l]SJ[n,l](37c)×PJB,nullH[n,l])≤PJ,(37d)CB(Qs[n,l])−ηFBL≥RBmin,(37e)fQs[n,l],SJ[n,l]≥RminFBL,(37f)Qs[n,l]⪰0,SJ[n,l]⪰0,(37g)rankQs[n,l]≤1,

where $R_{B}^{\min}$ is the minimum required rate at Bob and $R_{\min}^{\mathrm{FBL}}$ is the minimum required secrecy rate under FBL transmission. The constraint in (37b) limits Alice’s transmit power, the constraint in (37c) enforces Jamie’s AN power budget after null-space projection, the constraint in (37d) ensures a minimum quality-of-service (QoS) requirement at Bob, the constraint in (37e) guarantees a minimum FBL secrecy rate requirement, the constraints in (37f) ensure that the transmit and AN shaping covariance matrices are physically realizable, and the constraint in (37g) enforces a single-stream beamforming structure at Alice. Although the objective function has a difference-of-concave (DC) structure and is jointly non-concave in $\left(Q_{s}\left[n,l\right],S_{J}]n,l]\right)$, problem (P2) remains tractable. Since both the objective and all constraints are separable across frames, problem (P2) can be decomposed into NL independent per-frame subproblems.

### 5.3. Single-Frame Optimization Problem

For notational simplicity, the indices (n,l) are omitted, and all variables refer to the considered frame. The resulting problem is given by

(38a)(P3):maxQs,SJCB(Qs)−C˜E(Qs,SJ)−ηFBL(38b)s.t.tr(Qs)≤PA,(38c)             trPJB,nullSJPJB,nullH≤PJ,(38d)             CB(Qs)−ηFBL≥RBmin,(38e)             CB(Qs)−C˜E(Qs,SJ)−ηFBL≥RminFBL,(38f)             Qs⪰0,SJ⪰0,(38g)             rank(Qs)≤1.

Since problem (P3) inherits the non-convexity of problem (P2), we develop a solution that combines SDR, AO, and SCA. Specifically, the rank-one constraint on $Q_{s}$ is first relaxed via SDR, yielding a convex feasible set. The remaining non-convexity arises from the coupling between $Q_{s}$ and $S_{J}$ and the DC structure of the secrecy objective. To handle this, we adopt an AO framework in which $Q_{s}$ and $S_{J}$ are optimized in an alternating manner. In each iteration, one variable is optimized while the other is fixed, thereby decomposing the original problem into two more tractable subproblems. Within each AO step, the DC structure is addressed using SCA, where the concave component ${\tilde{C}}_{E}$ is approximated by its first-order Taylor expansion at the current operating point. This yields a global affine upper bound, ensuring that each subproblem is convex. For each frame, Algorithm 2 is applied to optimize the transmit covariance matrix and the AN shaping covariance matrix.
**Algorithm 2**  Inner-Layer Optimization for a Generic Frame**Require:** 
${\hat{R}}_{AE}$, ${\hat{R}}_{JE}$, $H_{AB}$, $H_{JB}$, $P_{A}$, $P_{J}$, $\sigma_{E}^{2}$, $\eta_{\mathrm{FBL}}$, $R_{B}^{\min}$, $R_{\min}^{\mathrm{FBL}}$, tolerance $\delta_{\mathrm{AO}}>0$, and maximum number of iterations $K_{\max}$.**Ensure:** 
Optimized transmit covariance $Q_{s}^{★}$ and AN covariance $Q_{J}^{★}$.1:Compute the null-space projection matrix $P_{JB,\mathrm{null}}$.2:Initialize feasible $Q_{s}^{\left(0\right)}⪰0$ and $S_{J}^{\left(0\right)}⪰0$ satisfying the constraints of (P3) (e.g., scaled identity matrices).3:Form $Q_{J}^{\left(0\right)}=P_{JB,\mathrm{null}}S_{J}^{\left(0\right)}P_{JB,\mathrm{null}}^{H}$.4:Evaluate the initial secrecy objective: $f^{\left(0\right)}=C_{B}\;\left(Q_{s}^{\left(0\right)}\right)-{\tilde{C}}_{E}\;\left(Q_{s}^{\left(0\right)},S_{J}^{\left(0\right)}\right)-\eta_{\mathrm{FBL}}$.5:Set k←0.6:**repeat**7:   **Transmit-covariance matrix update:**8:   Compute $\beta_{E}^{\left(k\right)}=\mathrm{tr}\;\left(Q_{J}^{\left(k\right)}{\hat{R}}_{JE}\right)+\sigma_{E}^{2}$.9:   Construct the affine upper bound ${\tilde{C}}_{E}^{\mathrm{ub},(k)}\left(Q_{s}\right)$ via first-order Taylor expansion.10:   Solve convex problem (P3.1) to obtain $Q_{s}^{\left(k+1\right)}$.11:   **AN shaping covariance matrix update:**12:   Compute $\psi^{\left(k+1\right)}=\mathrm{tr}\;\left(Q_{s}^{\left(k+1\right)}{\hat{R}}_{AE}\right)$.13:   Solve convex problem (P3.2) to obtain $S_{J}^{\left(k+1\right)}$.14:   Recover $Q_{J}^{\left(k+1\right)}=P_{JB,\mathrm{null}}S_{J}^{\left(k+1\right)}P_{JB,\mathrm{null}}^{H}$.15:   Evaluate the updated secrecy objective: $f^{\left(k+1\right)}=C_{B}\;\left(Q_{s}^{\left(k+1\right)}\right)-{\tilde{C}}_{E}\;\left(Q_{s}^{\left(k+1\right)},S_{J}^{\left(k+1\right)}\right)-\eta_{\mathrm{FBL}}.$16:   k←k+117:**until **$|f^{\left(k\right)}-f^{\left(k-1\right)}|\leq \delta_{\mathrm{AO}}$** or **$k=K_{\max}$18:Set $Q_{s}^{★}\leftarrow Q_{s}^{\left(k\right)}$ and $Q_{J}^{★}\leftarrow Q_{J}^{\left(k\right)}$.

At the *k*-th iteration, we first fix the AN shaping covariance matrix $S_{J}^{\left(k\right)}$, which determines the corresponding AN covariance matrix as $Q_{J}^{\left(k\right)}=P_{JB,\mathrm{null}}S_{J}^{\left(k\right)}P_{JB,\mathrm{null}}^{H}$. The resulting interference-plus-noise power at Eve is defined as $\beta_{E}^{\left(k\right)}≜\mathrm{tr}\;\left(Q_{J}^{\left(k\right)}{\hat{R}}_{JE}\right)+\sigma_{E}^{2}$. Accordingly, Eve’s achievable rate can be expressed as ${\tilde{C}}_{E}\left(Q_{s};S_{J}^{\left(k\right)}\right)=\log_{2}\;\left(1+\frac{\mathrm{tr}(Q_{s}{\hat{R}}_{AE})}{\beta_{E}^{\left(k\right)}}\right)$, which is concave in $Q_{s}$. To convexify the objective, we linearize ${\tilde{C}}_{E}\left(Q_{s};S_{J}^{\left(k\right)}\right)$ at the current iterate $Q_{s}^{\left(k\right)}$. The first-order Taylor expansion yields the following global upper bound:

(39) $${\tilde{C}}_{E}\left(Q_{s};S_{J}^{\left(k\right)}\right)\leq {\tilde{C}}_{E}\left(Q_{s}^{\left(k\right)};S_{J}^{\left(k\right)}\right)+\frac{\mathrm{tr}\;\left({\hat{R}}_{AE}\left(Q_{s}-Q_{s}^{\left(k\right)}\right)\right)}{\left(\beta_{E}^{\left(k\right)}+\mathrm{tr}\left(Q_{s}^{\left(k\right)}{\hat{R}}_{AE}\right)\right)\ln2}.$$

By denoting this affine surrogate by ${\tilde{C}}_{E}^{\mathrm{ub},(k)}\left(Q_{s}\right)$, the transmit covariance matrix is updated by solving the following subproblem:

(40a)(P3.1):maxQsCB(Qs)−C˜Eub,(k)(Qs)−ηFBL(40b)s.t.tr(Qs)≤PA,(40c)Qs⪰0,(40d)CB(Qs)−ηFBL≥RBmin,(40e)CB(Qs)−C˜Eub,(k)(Qs)−ηFBL≥RminFBL.

This subproblem is convex because $C_{B}\left(Q_{s}\right)$ is concave and ${\tilde{C}}_{E}^{\mathrm{ub},(k)}\left(Q_{s}\right)$ is affine.

For fixed $Q_{s}^{\left(k+1\right)}$, we update the AN shaping covariance matrix $S_{J}$. By defining $\psi^{\left(k+1\right)}≜\mathrm{tr}\;\left(Q_{s}^{\left(k+1\right)}{\hat{R}}_{AE}\right)$, Eve’s achievable rate can be expressed as ${\tilde{C}}_{E}\left(S_{J}\right)=\log_{2}\;\left(1+\frac{\psi^{\left(k+1\right)}}{\mathrm{tr}\;\left(Q_{J}{\hat{R}}_{JE}\right)+\sigma_{E}^{2}}\right)$.

Let $x\left(S_{J}\right)=\mathrm{tr}\;\left(Q_{J}{\hat{R}}_{JE}\right)+\sigma_{E}^{2}$. Since $Q_{J}=P_{JB,\mathrm{null}}S_{J}P_{JB,\mathrm{null}}^{H}$, $x(S_{J})$ is affine in $S_{J}$. The function $g\left(x\right)=\log_{2}\;\left(1+\frac{\psi^{\left(k+1\right)}}{x}\right)$ is convex and monotonically decreasing for x>0. Hence, by composition rules, ${\tilde{C}}_{E}\left(S_{J}\right)=g\left(x\left(S_{J}\right)\right)$ is convex. Accordingly, the AN shaping covariance update is obtained by solving the following subproblem:

(41a)(P3.2):minSJC˜E(SJ)(41b)s.t.trQJ≤PJ,(41c)CB(Qs(k+1))−C˜E(SJ)−ηFBL≥RminFBL,(41d)SJ⪰0,

where $Q_{J}=P_{JB,\mathrm{null}}S_{J}P_{JB,\mathrm{null}}^{H}$. This subproblem is convex since the objective function ${\tilde{C}}_{E}\left(S_{J}\right)$ is convex, and all constraints define a convex feasible set.

### 5.4. Beamformer and a Precoder Recovery

After solving the subproblems (P3.1) and (P3.2), the optimized transmit covariance $Q_{s}^{★}\left[n,l\right]$ and AN shaping covariance $S_{J}^{★}\left[n,l\right]$ are used to recover the corresponding beamforming and precoding matrices. If $\mathrm{rank}(Q_{s}^{★}\left[n,l\right])=1$, the optimal beamforming vector is obtained via eigenvalue decomposition as $Q_{s}^{★}\left[n,l\right]=w^{★}\left[n,l\right]{\left(w^{★}\left[n,l\right]\right)}^{H}$, where $w^{★}\left[n,l\right]$ is given by the principal eigenvector scaled by the square root of the dominant eigenvalue. However, if $\mathrm{rank}(Q_{s}^{★}\left[n,l\right])>1$, a feasible beamforming vector can be obtained using a dominant-eigenvector approximation or Gaussian randomization. In the latter case, candidate vectors are generated according to $\mathrm{CN}(0,Q_{s}^{★}\left[n,l\right])$, normalized to satisfy the transmit power constraint, and the one achieving the best objective value is selected. The AN covariance matrix is given by $Q_{J}^{★}\left[n,l\right]=P_{JB,\mathrm{null}}\left[n,l\right]S_{J}^{★}\left[n,l\right]P_{JB,\mathrm{null}}^{H}\left[n,l\right]$. To recover the AN precoder, we factorize $S_{J}^{★}\left[n,l\right]$. Let the eigendecomposition of $S_{J}^{★}\left[n,l\right]$ be $S_{J}^{★}\left[n,l\right]=U_{S}\left[n,l\right]\Lambda_{S}\left[n,l\right]U_{S}^{H}\left[n,l\right]$, where $U_{S}\left[n,l\right]\in C^{N_{J}\times d_{J}}$ contains the eigenvectors corresponding to the nonzero eigenvalues, $\Lambda_{S}\left[n,l\right]\in C^{d_{J}\times d_{J}}$ is a diagonal matrix with positive entries, and $d_{J}=\mathrm{rank}\left(S_{J}^{★}\left[n,l\right]\right)$ denotes the number of AN streams. A valid AN shaping matrix is obtained as ${\tilde{V}}^{★}\left[n,l\right]=U_{S}\left[n,l\right]\Lambda_{S}^{1/2}\left[n,l\right]$, and the corresponding AN precoder is constructed as $V^{★}\left[n,l\right]=P_{JB,\mathrm{null}}\left[n,l\right]{\tilde{V}}^{★}\left[n,l\right]$. This construction ensures that $Q_{J}^{★}\left[n,l\right]=V^{★}\left[n,l\right]{\left(V^{★}\left[n,l\right]\right)}^{H}$ and $H_{JB}\left[n,l\right]V^{★}\left[n,l\right]=0$, i.e., the AN lies entirely in the null space of the Jamie-Bob channel and does not interfere with the legitimate receiver.

### 5.5. Complexity of the Optimization Framework

Let $I_{\mathrm{bis}}$ denote the number of bisection iterations in the outer layer, which grows logarithmically with the accuracy requirement ϵ. Since each iteration requires an EKF covariance update with complexity $O(d_{x}^{3})$, where $d_{x}=3$ is the state dimension, the overall sensing-layer complexity is $O(NI_{\mathrm{bis}}d_{x}^{3})$. For the inner layer, let $I_{\mathrm{AO}}$ denote the number of AO iterations. Since $Q_{s}\in C^{N_{A}\times N_{A}}$ and $S_{J}\in C^{N_{J}\times N_{J}}$, the problem dimension scales as $n_{v}^{\mathrm{com}}=O\left(N_{A}^{2}+N_{J}^{2}\right)$, yielding a per-frame complexity of $O\;\left(I_{\mathrm{AO}}{\left(n_{v}^{\mathrm{com}}\right)}^{3.5}\right)$ based on standard interior-point methods [36]. As the communication design is performed over all NL frames, the total communication-layer complexity is $O\;\left(NLI_{\mathrm{AO}}{\left(n_{v}^{\mathrm{com}}\right)}^{3.5}\right)$. Therefore, the overall complexity is $O\;\left(NI_{\mathrm{bis}}d_{x}^{3}+NLI_{\mathrm{AO}}{\left(n_{v}^{\mathrm{com}}\right)}^{3.5}\right)$. In practice, the sensing layer incurs a much lower computational burden due to the low-dimensional EKF operations and scalar bisection search. Specifically, the outer-layer sensing optimization is executed online once per time slot to adapt the sensing resources according to the current EKF tracking performance, with an indicative runtime on the order of a few milliseconds under the considered system dimensions. In contrast, the inner-layer communication optimization is intended to operate as a slower-timescale controller that updates the transmit covariance matrices using the latest sensing information rather than performing a complete optimization for every transmitted packet. Its runtime depends strongly on the optimization solver, software implementation, and computing hardware. It is noted that the communication optimization is performed over low-dimensional covariance matrices when the number of antennas is not large, resulting in relatively small semidefinite programs that converge within a limited number of AO iterations. In addition, the communication optimization problems corresponding to different frames (n,l) are independent and can therefore be executed in parallel on multi-core processors or edge-computing platforms. Consequently, the proposed two-time-scale optimization framework is well suited for implementation on modern vehicular on-board processors or edge-assisted computing infrastructures under the considered system dimensions. Nevertheless, larger antenna arrays, denser network deployments, or more stringent latency requirements may require lightweight approximation algorithms or AI-assisted optimization methods to further reduce the computational burden, which constitutes an interesting direction for future research.

## 6. Results and Discussion

This section presents MATLAB R2025b simulation results obtained using the CVX toolbox with the SDPT3 solver on a desktop computer equipped with an Intel Core i7-13700 processor (Intel Corporation, Santa Clara, CA, USA) and 32 GB of RAM to evaluate the performance of the proposed framework. The main simulation parameters are listed in Table 3. A dynamic bidirectional vehicular scenario is considered in a two-dimensional (2-D) Cartesian plane, where Alice and Eve move along the positive *x*-axis, while Bob and Jamie move in the opposite direction. The node positions evolve according to $x_{i}\left[n+1\right]=x_{i}\left[n\right]+v_{i}\left[n\right]\tau$ and $y_{i}\left[n+1\right]=y_{i}\left[n\right]$ with i∈{A,B,E,J}. Then, the distance between any two nodes *i* and *j* is computed as $d_{ij}\left[n\right]=\sqrt{{\left(x_{i}\left[n\right]-x_{j}\left[n\right]\right)}^{2}+{\left(y_{i}\left[n\right]-y_{j}\left[n\right]\right)}^{2}}$. All azimuth angles are measured with respect to the positive *x*-axis, which coincides with the ULA broadside direction. The cluster AoD/AoA angles follow a Laplacian distribution centered at the geometric LoS azimuth angle $\theta_{ij}^{\mathrm{LoS}}\left[n\right]=atan2\left(y_{j}\left[n\right]-y_{i}\left[n\right],x_{j}\left[n\right]-x_{i}\left[n\right]\right)$ with a standard deviation of $5^{\circ }$. The initial EKF state is set as ${\hat{x}}_{JE}\left[1|0\right]={\left[\theta_{JE}^{\mathrm{LoS}}\left[1\right],\;d_{JE}\left[1\right],\;v_{E}-v_{J}\right]}^{T}$, where $v_{E}-v_{J}$ denotes the relative longitudinal velocity. Half-wavelength antenna spacing is assumed, while $\epsilon_{JE}\left[n\right]$ is generated as a circularly symmetric complex Gaussian random variable with zero mean and unit variance [30]. Unless otherwise stated, the regularization coefficient is $c_{r}=10^{-3}$, the bisection tolerance is $\epsilon =10^{-6}$, and the AO algorithm terminates when the objective improvement falls below $\delta_{\mathrm{AO}}=10^{-3}$ or after $K_{\max}=20$ iterations. For rank-one recovery, Gaussian randomization with 50 trials is employed when $\mathrm{rank}(Q_{s})>1$, and the candidate yielding the highest secrecy rate is selected. All simulation results are obtained by averaging over 100 independent Monte Carlo realizations. For each realization, the proposed framework and all benchmark schemes are evaluated using identical channel coefficients, mobility trajectories, process-noise realizations, and measurement-noise realizations to ensure a fair comparison. Independent pseudo-random number generator seeds are employed across different Monte Carlo realizations, while identical realizations are reused across all compared schemes within each run. Under the aforementioned simulation setup and convergence criteria, the outer-layer sensing optimization required an average execution time of 3.2 ms per time slot, while the inner-layer communication optimization required an average execution time of 1.28 s per frame-level optimization instance. The AO procedure converged in an average of 8.6 iterations. To evaluate the practical tightness of the SDR relaxation, the rank of the optimized transmit covariance matrix was monitored throughout the Monte Carlo simulations. The relaxed solution was rank one in 92.5% of the optimization instances, allowing direct recovery of the transmit beamforming vector via eigenvalue decomposition. Consequently, Gaussian randomization was required in only 7.5% of the cases. The average secrecy-rate degradation after recovering a feasible beamforming vector was below 1.0% relative to the relaxed SDR solution, confirming that the SDR relaxation is near-tight under the considered system dimensions.

The adopted benchmark schemes are designed to isolate the contribution of the individual components of the proposed framework, while their design is motivated by representative secure ISAC and robust beamforming methodologies reported in the recent literature [4,9,26]. Specifically, the proposed framework is compared with: (i) a snapshot-based benchmark that performs sensing independently at each slot without recursive EKF prediction, reflecting sensing-assisted secure beamforming approaches that rely on instantaneous sensing information [9,26]; (ii) a non-robust benchmark that neglects uncertainty-aware covariance construction and performs beamforming using only the estimated eavesdropper direction, representing nominal secure beamforming designs that optimize transmission based on estimated spatial information without explicitly accounting for estimation uncertainty; (iii) a fixed null-space AN benchmark employing conventional isotropic AN within the null space of the legitimate channel, representing null-space jamming designs that protect the legitimate receiver from AN leakage but do not adaptively shape the AN covariance according to the eavesdropper uncertainty; (iv) a jamming-without-null-space benchmark that transmits AN without null-space projection, representing conventional AN-assisted secure transmission in which jamming may also introduce interference leakage toward the legitimate receiver; and (v) a no-jamming benchmark that disables cooperative jamming and relies solely on sensing-assisted communication optimization, consistent with secure ISAC designs without dedicated AN-assisted cooperative jamming [4]. It is acknowledged that these benchmarks represent literature-inspired methodological counterparts and internal ablations rather than exact implementations of previously published robust secure ISAC frameworks. This limitation arises because, to the best of our knowledge, no existing method jointly considers mobility-aware recursive tracking, uncertainty-aware covariance construction, cooperative jamming, and FBL secrecy optimization under the same dynamic V2V system assumptions, making a direct one-to-one comparison under identical settings not currently feasible.

**Figure 3:**
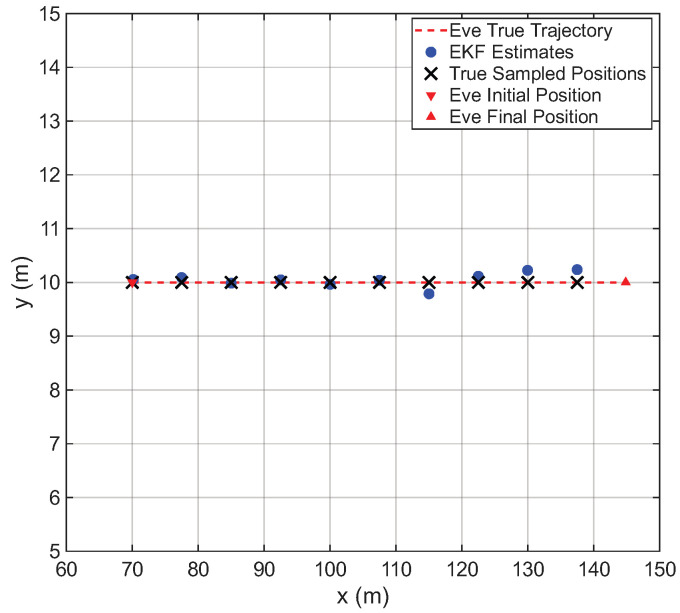
True and EKF-estimated trajectories of Eve.

Figure 3 compares the true and EKF-estimated trajectories of Eve. Eve moves along a straight-line road trajectory according to the adopted vehicular mobility model, while Jamie continuously performs ISAC-enabled sensing and recursively updates Eve’s state estimate through the EKF prediction and update operations. The estimated positions closely follow the true trajectory over the entire observation interval, demonstrating the effectiveness of the proposed mobility-aware sensing and tracking approach. Minor estimation errors arise from sensing noise, process uncertainty, limited sensing resources, and mobility-induced prediction errors. Nevertheless, the EKF maintains accurate and stable tracking performance, effectively capturing the temporal evolution of Eve’s trajectory. To provide a quantitative assessment of the EKF tracking accuracy, Monte Carlo simulations were conducted over 100 independent realizations using different process and measurement noise sequences. Table 4 summarizes the time-averaged root-mean-square errors (RMSEs) of the estimated angular direction, distance, relative velocity, and two-dimensional position, together with their corresponding 95% confidence intervals (CIs). The consistently low RMSE values and narrow confidence intervals confirm the effectiveness and robustness of the proposed EKF-based tracking algorithm. These quantitative results complement the representative trajectory shown in Figure 3 and demonstrate that the EKF provides reliable uncertainty-aware covariance information for the subsequent robust beamforming and AN design in dynamic vehicular environments.

**Figure 4:**
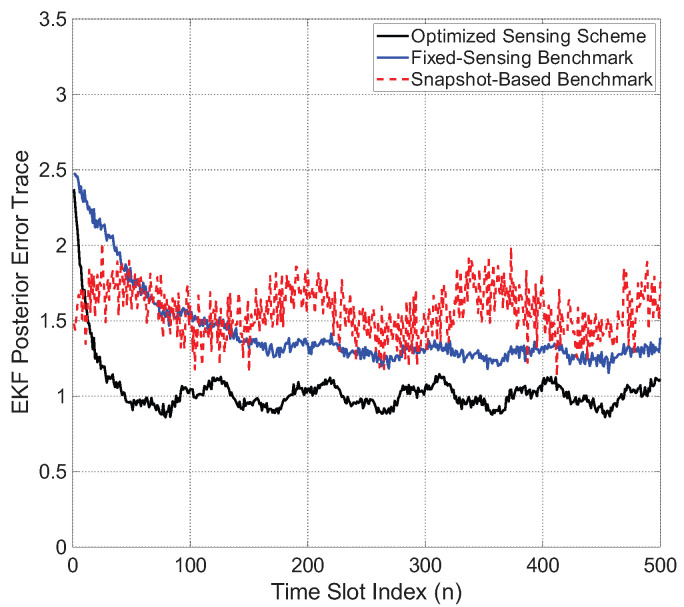
Evolution of the EKF posterior error covariance trace.

Figure 4 illustrates the evolution of the EKF posterior error covariance trace $\mathrm{tr}\left(P[n|n]\right)$ over the considered observation horizon. The proposed sensing optimization rapidly reduces the initial tracking uncertainty and subsequently maintains the covariance close to the target threshold $\delta_{P}$ through adaptive sensing-resource allocation, resulting in improved estimation accuracy and tracking stability. In contrast, the fixed-sensing benchmark employs a non-adaptive sensing configuration in which the sensing duration and sensing energy remain unchanged regardless of the instantaneous tracking conditions. Although recursive EKF-based tracking is still utilized, the inability to dynamically adjust the sensing resources results in higher steady-state covariance levels compared to the proposed optimized scheme. Furthermore, the snapshot-based benchmark exhibits the highest covariance values together with more pronounced irregular fluctuations, since it performs independent slot-wise sensing-based estimation without exploiting recursive EKF state prediction, temporal memory, or prior covariance information.

**Figure 5:**
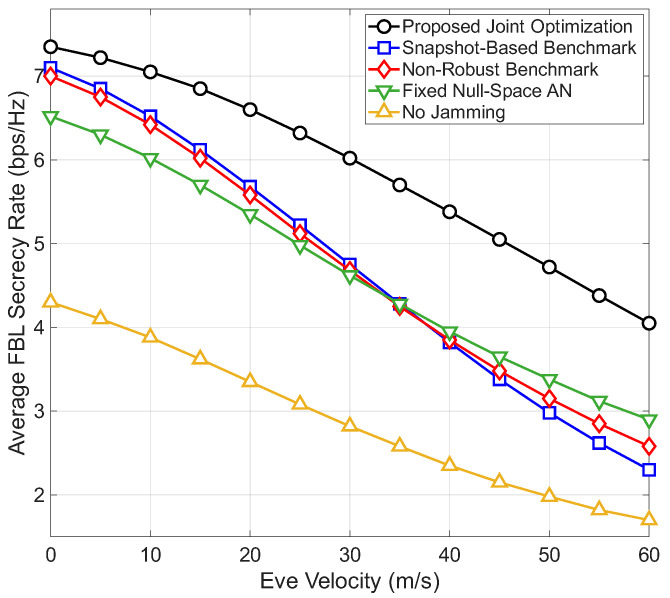
Average FBL secrecy rate versus Eve velocity under different sensing, tracking, and communication strategies.

Figure 5 evaluates the robustness of the proposed framework under increasing Eve mobility for different benchmark schemes. As Eve’s velocity increases, the average FBL secrecy rate decreases for all schemes because higher mobility leads to larger state-prediction errors, faster spatial channel variations, and reduced tracking accuracy. This increases the mismatch between Eve’s actual direction and the uncertainty-aware covariance information used for beamforming and AN design. Nevertheless, the proposed EKF-assisted joint optimization consistently achieves the highest secrecy rate over the entire velocity range, demonstrating its robustness against mobility-induced uncertainty. This gain comes from the recursive EKF prediction-update mechanism, which exploits temporal state correlation and continuously refines the posterior covariance matrix used in the spatial covariance construction. In contrast, the snapshot-based benchmark relies only on instantaneous sensing observations and therefore suffers more severe degradation at high velocities. The non-robust benchmark is more sensitive to sensing mismatch, while the fixed null-space AN benchmark is limited by its lack of adaptive AN shaping.

**Figure 6:**
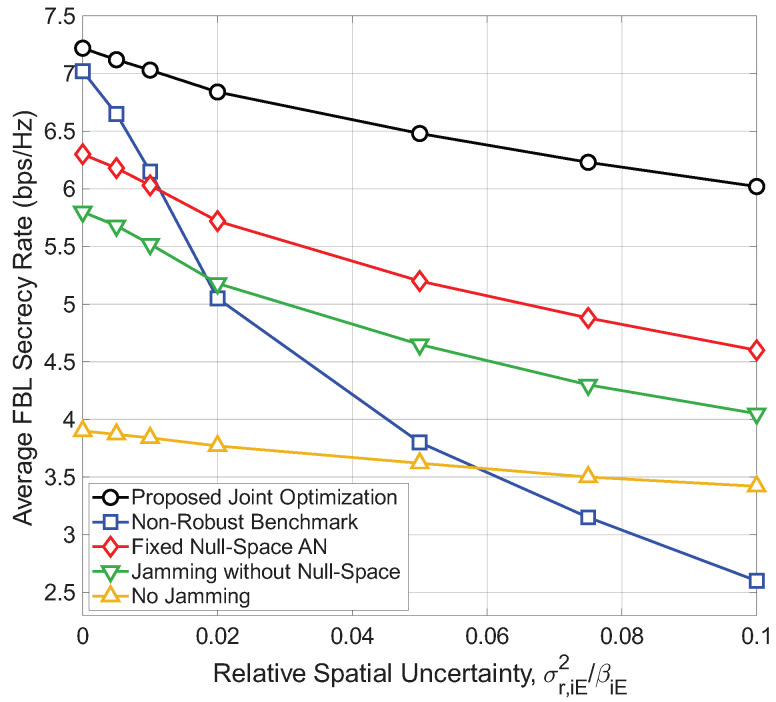
Average FBL secrecy rate versus the relative sensing-induced spatial uncertainty $\sigma_{r,iE}^{2}/\beta_{iE}$.

Figure 6 investigates the impact of sensing-induced spatial uncertainty on the achievable FBL secrecy rate. The uncertainty level is represented through the diagonal-loading variance $\sigma_{r,iE}^{2}$, which models isotropic spatial spreading in the uncertainty-aware covariance matrices. As the uncertainty increases, the covariance matrices toward Eve become less directional, reducing the effectiveness of transmit beamforming and AN spatial focusing. Hence, the secrecy performance of all schemes degrades. However, the uncertainty-aware framework maintains the highest secrecy rate, since the diagonal-loading term explicitly captures residual angular uncertainty and diffuse scattering effects. In contrast, the fixed null-space AN benchmark suffers a stronger performance loss because its AN covariance is not adaptively shaped. The non-null-space jamming benchmark is further limited by AN leakage toward Bob, while the no-jamming benchmark provides the lowest secrecy performance due to the absence of AN transmission. On the other hand, the non-robust benchmark performs well under very small uncertainty levels, where the assumed angular direction is nearly accurate, but degrades rapidly as uncertainty increases.

**Figure 7:**
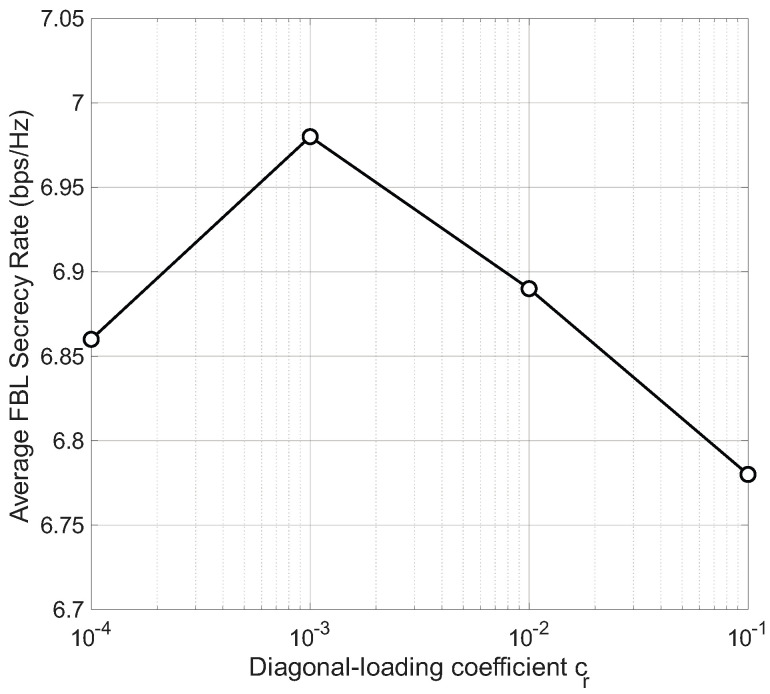
Average FBL secrecy rate versus the diagonal-loading coefficient $c_{r}$.

Figure 7 evaluates the sensitivity of the proposed framework to the diagonal-loading coefficient $c_{r}$. Smaller values of $c_{r}$ produce highly directional covariance matrices that are more susceptible to angular estimation errors, whereas excessively large values introduce an overly diffuse uncertainty component and reduce the effectiveness of spatially selective beamforming and AN transmission. Nevertheless, moderate variations around the selected value $c_{r}=10^{-3}$ result in only minor changes in the achieved average FBL secrecy rate. This demonstrates that the proposed framework is robust to the choice of the diagonal-loading coefficient and that the selected value provides a suitable tradeoff between beamforming directivity and robustness against angular uncertainty under the considered system parameters.

**Figure 8:**
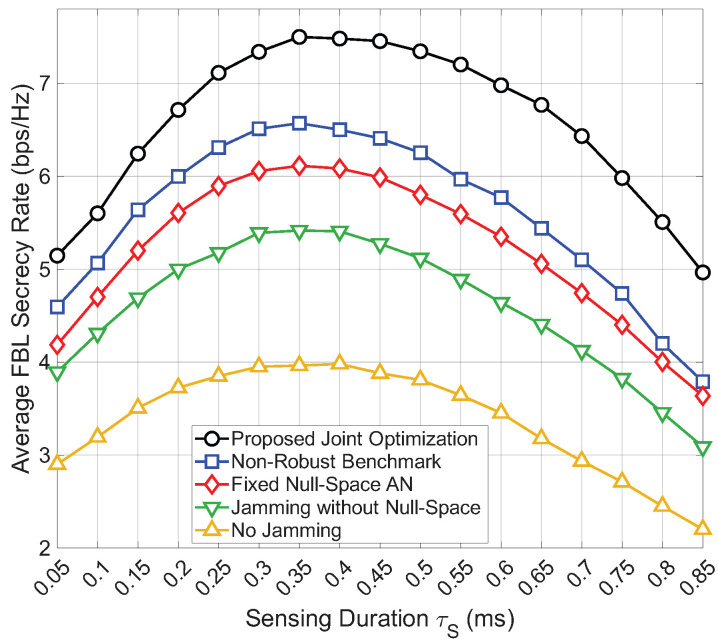
Average FBL secrecy rate versus sensing duration $\tau_{S}$.

Figure 8 shows the sensing-communication tradeoff under a fixed slot duration. As the sensing duration $\tau_{S}$ increases, the secrecy rate initially improves because longer sensing intervals reduce EKF estimation error and improve the quality of the uncertainty-aware covariance matrices. Improved sensing accuracy leads to more accurate spatial covariance construction and more effective beamforming and AN shaping. However, beyond a certain point, further increasing $\tau_{S}$ reduces the remaining communication time available within the slot, thereby degrading secrecy performance. The proposed uncertainty-aware framework consistently achieves the highest secrecy performance because it effectively balances sensing accuracy and communication efficiency through the joint optimization of sensing resources, transmit covariance, and AN shaping. In contrast, the benchmark schemes experience lower peak secrecy rates and sharper performance degradation at large sensing durations due to less efficient utilization of the sensing and communication resources.

**Figure 9:**
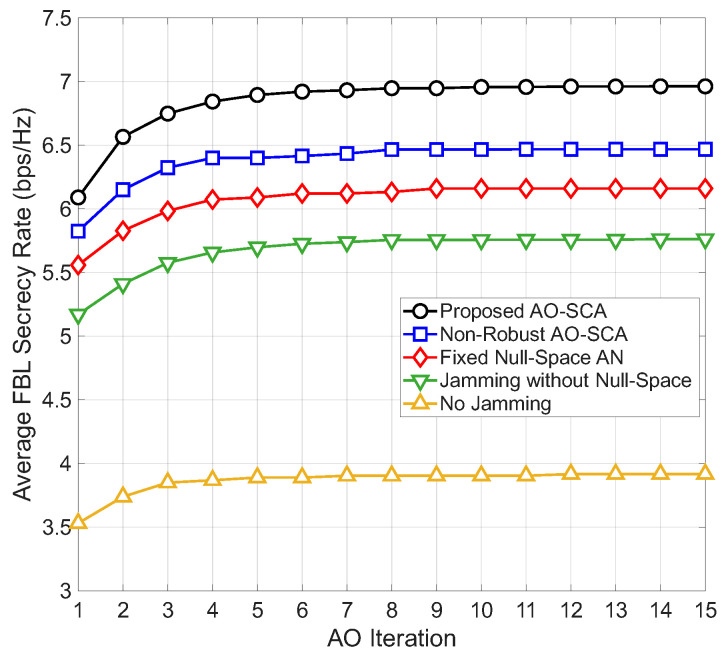
Inner-layer AO-SCA convergence behavior for the proposed and benchmark communication schemes.

Figure 9 depicts the convergence behavior of the proposed inner-layer AO-SCA optimization framework. The results show that the FBL secrecy rate rapidly converges to a stable value within a relatively small number of iterations. Among the considered schemes, the proposed method achieves the highest secrecy rate due to the joint optimization of the transmit covariance and AN shaping covariance. The fixed null-space AN benchmark converges to a lower secrecy rate because the AN covariance is not jointly optimized with the transmit covariance, but instead follows a predefined isotropic null-space structure. Similarly, the non-null-space jamming benchmark exhibits inferior secrecy performance since part of the AN leaks toward Bob, thereby degrading the legitimate communication link while attempting to jam Eve. The no-jamming benchmark achieves the lowest secrecy rate because only Alice’s transmit covariance is optimized, without employing AN to suppress Eve’s reception. Moreover, the non-robust benchmark, which assumes perfect angular knowledge and does not account for uncertainty-aware covariance construction, experiences noticeable performance degradation under sensing uncertainty compared to the proposed robust scheme.

**Figure 10:**
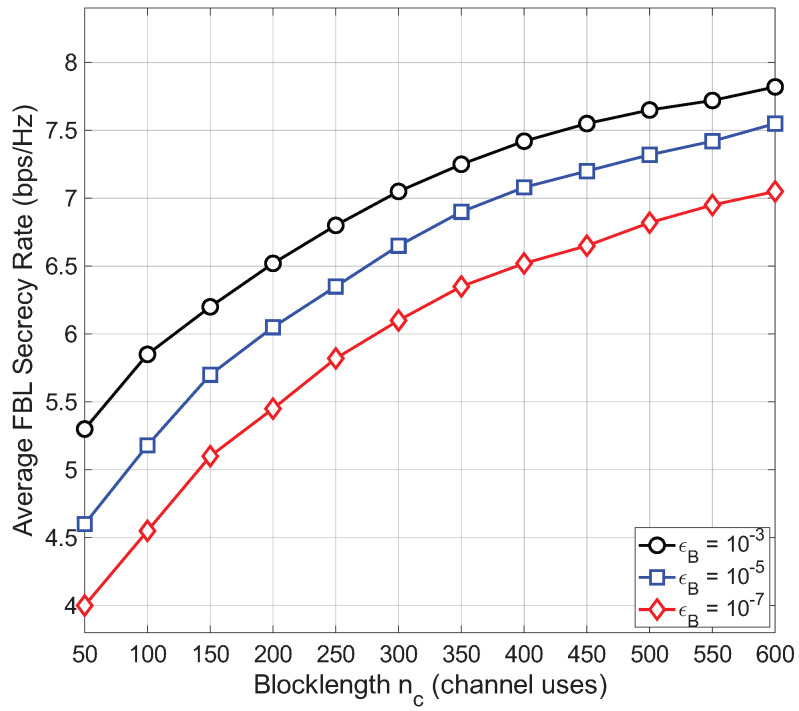
Average FBL secrecy rate versus blocklength $n_{c}$ for different decoding error probability requirements $\varepsilon_{B}$.

Finally, Figure 10 evaluates the impact of the blocklength $n_{c}$ and the decoding error probability requirement $\epsilon_{B}$ on the average FBL secrecy rate of the proposed inner-layer communication framework. The achievable secrecy rate increases with the blocklength because the FBL loss becomes smaller and the transmission progressively approaches the asymptotic Shannon-capacity regime. In particular, for small blocklengths, the FBL effect is significant, resulting in noticeable secrecy-rate degradation. However, as $n_{c}$ increases, the secrecy rate gradually saturates, indicating diminishing gains for very large blocklengths. Moreover, stricter reliability requirements lead to lower secrecy performance. Specifically, the case with $\epsilon_{B}=10^{-3}$ achieves the highest secrecy rate, whereas the more stringent URLLC reliability constraints $\epsilon_{B}=10^{-5}$ and $\epsilon_{B}=10^{-7}$ result in progressively lower secrecy rates. To quantify the statistical uncertainty of the secrecy-performance results, 95% CIs were computed over the 100 independent Monte Carlo realizations. Table 5 reports the average and maximum 95% CI half-widths across the operating points considered in Figure 5, Figure 6, Figure 8 and Figure 10. The narrow confidence intervals confirm the statistical stability of the reported average FBL secrecy-rate trends.

## 7. Conclusions and Future Research Directions

### 7.1. Conclusions

This paper investigated URLLC-enabled V2V communications in the presence of a passive VE within a mobility-aware ISAC framework. A hierarchical two-time-scale architecture was proposed, in which a VJ performs radar sensing and EKF-based tracking at the slot level, while the communication layer jointly optimizes the transmit covariance and null-space AN shaping covariance matrices at the frame level. Based on this scheme, a joint sensing-communication optimization framework was developed and solved using SDR, AO, and SCA. Simulation results demonstrated accurate VE tracking and robust estimation performance under vehicular mobility. The proposed adaptive sensing strategy achieved significantly lower EKF posterior covariance than fixed-sensing and snapshot-based benchmarks, while the uncertainty-aware communication design consistently improved secrecy performance under increasing mobility and sensing uncertainty. The results also revealed the sensing-communication tradeoff imposed by URLLC constraints, confirmed the fast convergence of the proposed AO-SCA algorithm, and showed that larger blocklengths and relaxed reliability requirements enhance the secrecy rate.

### 7.2. Future Research Directions

Several promising directions can further extend the proposed framework. Specifically, more advanced vehicular scenarios involving multiple coordinated or non-coordinated passive VEs can be investigated, leading to robust worst-case or probabilistic secrecy formulations under multi-adversary settings. Moreover, the impact of imperfect sensing and feedback mechanisms deserves further investigation, since sensing delays, quantization effects, and signaling overhead between sensing and communication modules may significantly degrade tracking accuracy, uncertainty-aware covariance construction, and overall secrecy performance. Another important direction is the extension toward distributed and network-level ISAC architectures, where multiple vehicles or infrastructure nodes cooperatively perform sensing, tracking, and secure transmission, thereby improving spatial diversity and robustness against eavesdropping. Finally, extending the proposed framework to multi-user vehicular scenarios with multiple legitimate receivers constitutes another promising research direction. Such an extension would require joint optimization of multi-user beamforming, user scheduling, inter-user interference management, and FBL secrecy constraints, enabling the investigation of network-wide secrecy performance in dynamic vehicular environments.

## Figures and Tables

**Table 1:** Main symbols and definitions.

Symbol	Definition
$Q_{s}\left[n,l\right]$	Transmit covariance matrix at Alice
$Q_{J}\left[n,l\right]$	Artificial-noise covariance matrix at Jamie
$S_{J}\left[n,l\right]$	Artificial-noise shaping covariance matrix before null-space projection
$P_{JB,\mathrm{null}}\left[n,l\right]$	Null-space projection matrix of the Jamie–Bob channel
${\hat{R}}_{AE}\left[n\right]$	Uncertainty-aware spatial covariance matrix of the Alice–Eve link
${\hat{R}}_{JE}\left[n\right]$	Uncertainty-aware spatial covariance matrix of the Jamie–Eve link
P[n|n]	EKF posterior error covariance matrix
P[n|n−1]	EKF predicted error covariance matrix
$\tau_{S}\left[n\right]$	Sensing duration in the *n*-th slot
$E_{S}\left[n\right]$	Sensing energy in the *n*-th slot
$n_{c}$	Finite blocklength (channel uses per packet)
$R_{s}^{\mathrm{lb}}\left[n,l\right]$	Lower bound on the achievable FBL secrecy rate
$\sigma_{\theta ,iE}^{2}\left[n\right]$	Angular uncertainty variance toward Eve
$\delta_{P}$	Maximum allowable EKF tracking-error threshold

**Table 2:** Comparison of recent relevant works.

Reference	Optimization Objective	ISAC	URLLC	PLS	Mobility
Wang et al. 2025 [4]	Secrecy-rate maximization under sensing–communication coupling	✓	✗	✓	✗
Su et al., 2024 [9]	Joint secrecy-rate and CRB optimization for sensing-assisted PLS	✓	✗	✓	✗
Zhao et al., 2024 [11]	Joint beamforming and scheduling for periodic and aperiodic traffic	✓	✓	✗	✗
Wei et al., 2025 [12]	Sensing latency minimization in V2X systems	✓	✓	✗	Partial
Liu et al., 2026 [13]	CRB-rate tradeoff optimization in FBL MIMO systems	✓	✓	✗	✗
Katwe et al., 2025 [14]	Worst-case rate maximization in mBRLLC	✓	✓	✗	✗
Osouli et al., 2026 [15]	DRL-based sum-rate maximization in BD-RIS-assisted systems	✓	✓	✗	✗
Nguyen et al., 2024 [16]	Minimum secrecy-rate maximization	✗	✓	✓	✗
Pradhan et al., 2024 [17]	Secure energy-efficiency maximization in IIoT systems	✗	✓	✓	✗
Pradhan et al., 2026 [18]	Joint blocklength and power optimization in FD-CNOMA systems	✗	✓	✓	Partial
Xie et al., 2023 [19]	AESR maximization via channel training and AN design	✗	✓	✓	✗
Asad et al., 2025 [20]	Secure FL framework for vehicular communications	✗	✓	✓	✓
Nan et al., 2025 [21]	Fairness-aware secrecy beamforming with AN	✓	✗	✓	✗
Salem et al., 2026 [22]	Robust secrecy optimization for RSMA-active RIS-assisted systems	✓	✗	✓	✗
Chen et al., 2024 [23]	Joint secrecy-rate and sensing-power maximization	✓	✗	✓	✗
Chen et al., 2026 [24]	Weighted sum secrecy-rate maximization in cell-free systems	✓	✗	✓	✗
Illi et al., 2026 [25]	Sensing-performance maximization under secrecy and power constraints	✓	✗	✓	✗
Cao et al., 2025 [26]	Leakage minimization in sensing-aided secure communication	✓	✗	✓	✗
Xia et al., 2026 [27]	Secure beamforming with dedicated sensing signals	✓	✗	✓	Partial
Zhu et al., 2026 [28]	Unified secrecy-rate optimization under sensing constraints	✓	✗	✓	✗
**This Paper**	**Secrecy-rate maximization with mobility-aware sensing and tracking**	✓	✓	✓	✓

**Table 3:** Simulation Parameters.

System Parameters	Value
Number of time slots: *N*	500
Time slot duration: τ	0.01 s [30]
Number of frames per slot: *L*	10
**Vehicular Mobility and Geometry**	**Value**
Initial Alice position $\left(x_{A}\left[1\right],y_{A}\left[1\right]\right)$	(30,0) m
Initial Bob position $\left(x_{B}\left[1\right],y_{B}\left[1\right]\right)$	(50,0) m
Initial Eve position $\left(x_{E}\left[1\right],y_{E}\left[1\right]\right)$	(70,10) m
Initial Jamie position $\left(x_{J}\left[1\right],y_{J}\left[1\right]\right)$	(0,0) m
Initial longitudinal velocities $\left(v_{A}\left[1\right],v_{B}\left[1\right],v_{E}\left[1\right],v_{J}\left[1\right]\right)$	(+5,−5,+15,−5) m/s
**Channel Parameters**	**Value**
Alice transmit power: $P_{A}$	23 dBm
Jamie AN transmit power: $P_{J}$	23 dBm
Noise power: $\sigma^{2}=\sigma_{B}^{2}=\sigma_{E}^{2}$	−94 dBm
Symbol duration: $T_{\mathrm{sym}}$	0.1 μs
Number of antennas: $\left(N_{A},N_{B},N_{J}\right)$	(2,2,4)
Channel gain at 1 m: $\beta_{0}$	−53.57 dB [37]
Path-loss exponent: α	1.77 [37]
Number of clusters: $C_{ij}$	3
Cluster angular standard deviation	$5^{\circ }$
**URLLC Parameters**	**Value**
Blocklength: $n_{c}$	200 channel uses
Maximum transmission latency: $\tau_{\max}^{\mathrm{trans}}$	1 ms
Decoding error probability: $\varepsilon_{B}$	$10^{-5}$ [17]
Information leakage probability: $\varepsilon_{s}$	$10^{-2}$ [17]
Minimum Bob rate: $R_{B}^{\min}$	2 bps/Hz
Minimum secrecy rate: $R_{\min}^{\mathrm{FBL}}$	1 bps/Hz
**Sensing and EKF Parameters**	**Value**
Maximum sensing power: $P_{S,J}^{\max}$	20 dBm
Radar receiver noise power: $\sigma_{r}^{2}$	−94 dBm
Estimator constants: $\left(a_{\theta },a_{d},a_{v}\right)$	$\left(1.05\times 10^{-2},\;3.5\times 10^{-2},\;1.05\times 10^{-2}\right)$ [30]
Sensing symbol duration: $T_{\mathrm{sym},S}$	71.429μs [38]
Array gain factor: κ	2
Beamforming gain: $|\delta_{JE}{|}^{2}$	1 [29]
Process-noise covariance: $Q_{w}$	$\mathrm{diag}(0.02^{2},\;0.2^{2},\;0.5^{2})$ [39]
Initial EKF covariance: P[1|0]	$\mathrm{diag}(10^{-2},\;1,\;1)$
Tracking threshold: $\delta_{P}$	1

**Table 4:** Monte Carlo Evaluation of the EKF Tracking Accuracy.

State Variable	Average RMSE	Unit	95% CI
Angular direction $\theta_{JE}$	0.31	∘	[0.27,0.35]
Distance $d_{JE}$	0.39	m	[0.34,0.45]
Relative velocity $v_{JE}$	0.24	m/s	[0.20,0.29]
2-D position	0.52	m	[0.46,0.59]

**Table 5:** Statistical uncertainty of the main secrecy-performance results.

Performance Evaluation	Average 95% CI Half-Width (bps/Hz)	Maximum 95% CI Half-Width (bps/Hz)
Eve mobility (Figure 5)	0.12	0.18
Spatial uncertainty (Figure 6)	0.11	0.16
Sensing duration (Figure 8)	0.09	0.14
FBL parameters (Figure 10)	0.08	0.12

## Data Availability

The original contributions presented in this study are included in the article.

## Abbreviations

The following abbreviations are used in this manuscript:

AI	Artificial Intelligence
AESR	Achievable Effective Secrecy Rate
AN	Artificial Noise
AO	Alternating Optimization
BCD	Block Coordinate Descent
BD-RIS	Beyond-Diagonal Reconfigurable Intelligent Surface
CI	Confidence Interval
CRB	Cramér–Rao Bound
CSI	Channel State Information
CVX	Convex Optimization Toolbox
DC	Difference-of-Concave
DCE	Discriminatory Channel Estimation
DRL	Deep Reinforcement Learning
EKF	Extended Kalman Filter
FBL	Finite-Blocklength
FD-CNOMA	Full-Duplex Cooperative Non-Orthogonal Multiple Access
FL	Federated Learning
IIoT	Industrial Internet of Things
IoT	Internet of Things
IoV	Internet of Vehicles
ISAC	Integrated Sensing and Communication
LoS	Line-of-Sight
mBRLLC	Mobile Broadband Reliable Low-Latency Communication
Meta-PPO	Meta-Proximal Policy Optimization
MIMO	Multiple-Input Multiple-Output
MISO	Multiple-Input Single-Output
mmWave	Millimeter-Wave
PLS	Physical-Layer Security
POMDP	Partially Observable Markov Decision Process
PSD	Positive Semidefinite
QoS	Quality of Service
RCS	Radar Cross-Section
RIS	Reconfigurable Intelligent Surface
RMSE	Root-Mean-Square Error
RSMA	Rate-Splitting Multiple Access
SCA	Successive Convex Approximation
SCNR	Signal-to-Clutter-plus-Noise Ratio
SCP	Sequential Convex Programming
SDP	Semidefinite Programming
SDR	Semidefinite Relaxation
SEE	Secure Energy Efficiency
SINR	Signal-to-Interference-plus-Noise Ratio
SNR	Signal-to-Noise Ratio
ULA	Uniform Linear Array
URLLC	Ultra-Reliable Low-Latency Communication
V2V	Vehicle-to-Vehicle
V2X	Vehicle-to-Everything
VE	Vehicular Eavesdropper
VJ	Vehicular Jammer
